# Comparison of COVID-19 Resilience Index and Its Associated Factors across 29 Countries during the Delta and Omicron Variant Periods

**DOI:** 10.3390/vaccines10060940

**Published:** 2022-06-13

**Authors:** Le Duc Huy, Chung-Liang Shih, Yao-Mao Chang, Nhi Thi Hong Nguyen, Phan Thanh Phuc, Tsong-Yih Ou, Chung-Chien Huang

**Affiliations:** 1 Health Personnel Training Institute, University of Medicine and Pharmacy, Hue University, Hue 49120, Vietnam; leduchuy@hueuni.edu.vn (L.D.H.); nthnhi@hueuni.edu.vn (N.T.H.N.); 2 School of Health Care Administration, College of Management, Taipei Medical University, Taipei 10675, Taiwan; cjym@tmu.edu.tw (Y.-M.C.); phuc.pt@umc.edu.vn (P.T.P.); 3 Ministry of Health and Welfare, Taipei 115204, Taiwan; md01@mohw.gov.tw; 4 Research Center of Health and Welfare Policy, Taipei Medical University, Taipei 11031, Taiwan; 5 International Ph.D. Program in Biotech and Healthcare Management, College of Management, Taipei Medical University, Taipei 10675, Taiwan; 6 Department of Social Work, University Medical Center, Ho Chi Minh City 70000, Vietnam; 7 Division of Infectious Diseases, Department of Internal Medicine, Wan Fang Hospital, Taipei Medical University, Taipei 11696, Taiwan; 93023@w.tmu.edu.tw; 8 Department of Nursing, Cardinal Tien Junior College of Healthcare and Management, New Taipei 23143, Taiwan; 9 Department of Long-Term Care and School of Gerontology Health Management, College of Nursing, Taipei Medical University, Taipei 11031, Taiwan; 10 Department and School of Pharmacy, College of Pharmacy, Taipei Medical University, Taipei 11031, Taiwan; 11 Department of Medical Quality, Taipei Municipal Wan Fang Hospital, Taipei Medical University, Taipei 11696, Taiwan

**Keywords:** COVID-19, vaccine, variants, Delta, Omicron, NPIs

## Abstract

Our study aims to compare the pandemic resilience index and explore the associated factors during the Delta and Omicron variant periods. In addition, the study aims to identify the characteristics of countries that had good performances. We analyzed observation data among 29 countries over the first eight weeks during the two periods of Delta and Omicron variant dominance. Data were extracted from open public databases. The Omicron variant caused a lowered mortality rate per 100,000 COVID-19 patients; however, it is still imposing a colossal burden on health care systems. We found the percentage of the population fully vaccinated and high government indices were significantly associated with a better resilience index in both the Delta and Omicron periods. In contrast, the higher death rate of cancers and greater years lived with disability (YLD) caused by low bone density were linked with poor resilience index in the Omicron periods. Over two periods of Delta and Omicron, countries with good performance had a lower death rate from chronic diseases and lower YLD caused by nutrition deficiency and PM2.5. Our findings suggest that governments need to keep enhancing the vaccine coverage rates, developing interventions for populations with chronic diseases and nutrition deficiency to mitigate COVID-19 impacts on these targeted vulnerable cohorts.

## 1. Introduction

The COVID-19 pandemic is continuously imposing colossal burden on mortality and morbidity across countries even after vaccines were rolled out [1]. As of 21 May 2022, there are nearly 521 million confirmed cases recorded and more than 6.2 million deaths worldwide [2]. During the first strike of the pandemic, governments successfully curtailed the pandemic using different non-pharmaceutical interventions (NPIs) (e.g., social distancing, border control, contact tracing, test screening, etc.) while waiting for the development and rollout of the vaccine [3]. After one year of various vaccine programs being launched, governments actively implemented massive vaccination campaigns to reach the target of herd immunity. Since the vaccines have shown effectiveness in preventing deaths and alleviating severity, many governments have switched their policy from zero COVID to an adaptation of this in the new situation [4]. However, with the emergence of newer variants, several countries with a relatively high vaccination coverage suffered from new waves of the pandemic [5,6]. Recently, scientific evidence showed that the new variants (especially Omicron) have increased transmission speed and decreased vaccine efficacy [7]. Few large-scale public health studies have been available so far, while the diseases are widespread globally, national public health leaders are desperate to seek guidance. This study finds that tracking pandemic indicators remains an utmost vital task that supports policy makers to navigate their response in an effective and timely manner. Due to a large amount of underreporting and undetectable cases, the hospital bed occupancy rate, ICU occupancy rate, and mortality rate would provide a deeper more accurate insight into the soaring effects of the pandemic on society and health care. Based on the aforementioned indicators, Coccia recently developed a resilience index to measure as well as compare the performance of countries in controlling the pandemic [8].

On the other hand, evaluating the impact of NPIs, vaccine deployments, and other factors associated with pandemic management is also essential to support effective policy response. Many studies have shown the effectiveness of NPIs and vaccines on containing infection growth rates [9,10,11]. However, in the later pandemic stage, some countries reported challenges with vaccine hesitancy and low compliance with NPIs in their society [12,13]. This led to slowing down the speed of vaccination coverage and threatening the achievement of pandemic control. In addition, more recent evidence indicated that vaccine coverage, socio-economic factors, healthcare preparedness [14], government performance [8,15], and environmental factors (e.g., PM2.5) could play an important role in explaining the variation of COVID-19 cases and deaths [16]. Hence, examining how these factors influence the resilience index is crucial to current public health.

Although previous studies investigated the role of these socio-economic factors, there is a lack of studies accounting for the emergence of new variants, particularly Omicron. Some studies from national databases showed that Omicron caused less severe symptoms than Delta [5,17]; however, there is little knowledge comparing Omicron and Delta variant’s impacts among countries. Therefore, in this study, we aimed to:

Firstly, compare the pandemic resilience index and explore the factors influencing the pandemic indicators during the Delta and Omicron periods.

Secondly, based on comparing the resilience index between the two periods, we also explore which countries have worse, medium, and good performance as well as examining their characteristics.

## 2. Materials and Methods

### 2.1. Study Design

We conducted a comparative study among 29 countries over the Delta and Omicron dominance periods.

Our study consisted of countries that provided the available data in terms of (a) prevalence of variant of concern on GISAID^®^ databases [18], (b) daily deaths, (c) daily hospital occupancy rate, and (d) daily ICU occupancy rate on the Our World^®^ in Data [19].

### 2.2. Variables

The outcome variable of the study is the resilience index of the specific country in a given period. According to Coccia, the resilience (*r*) index was calculated by the following formula [8]:

$$r_{j}=\overset{3}{\underset{i=1}{\sum }}\frac{F_{ij}}{3}\;with\;r_{j}<1;\;j=1,\ldots ,\;n\;countries;i=1,2,3\;$$

where

*F*_1*j*_ = The average daily mortality rate per 100,000 inhabitants in a specific country over a given period. The mortality rate refers to the daily number of deaths over the population in a particular country.

*F*_2*j*_ = The average daily hospital occupancy rate per 100,000 inhabitants in a particular country in a given period.

*F*_3*j*_ = The average daily ICU occupancy rate per 100,000 inhabitants in a particular country in a given period.

We standardized the values *F*_1*j*,_ *F*_2*j*,_ and *F*_3*j*_ from 0 to 1 before calculating the resilience index.

In this study, we calculated two kinds of *r* index: Weekly *r* index for longitudinal analysis and *r* index of the whole eight weeks in each variant period.

Data of daily mortality rate, hospital occupancy rate, and ICU occupancy rate are collected from Our World in Data [19]

The independent variables comprise different kinds of subgroup:

- COVID-19 interventions were measured by two indicators including stringency index and vaccine coverage. Stringency index measures the strictness of government response in terms of containment and closure policy [20]. We retrieved the vaccine data from the global vaccine databases, which consists of the percentage of population fully vaccinated, the percentage of population vaccinated with the booster dose [21].
- Sociodemographic variables: population density, percentage of population living in urban areas, the percentage of population aged over 65 years, the GDP per capita. These data were extracted from World Bank indicators [22].
- Government performance: government effectiveness index, government rule index, government quality index [22].
- Population characteristics related to health: death rate of chronic diseases per 100,000 inhabitants, years lived with disability (YLD), health behavior and environment risks. Data were retrieved from the global burden of disease databases [23].
- Healthcare capacities: global health security index, universal health coverage (UHC) index, hospital beds per 100,000, health care worker density, healthcare expenditure. Data were extracted from the World Health Organization, GHS.org [22,24,25].

Table S7 provides the definition of all variables and data sources.

### 2.3. Study Periods

To compare the resilience index among 29 countries between the Delta and the Omicron periods, we extracted weekly data on the time variance of variant prevalence in each country.

The period of variant dominance was recorded as the week when the variant prevalence was over 50% and followed up for eight weeks [26]. We selected eight weeks since it was the optimal period for gathering data from 29 nations as of the data collection date (20 March 2022) and assessing the effectiveness of government responses when new variants have emerged.

Finally, the study included 232 country-week observations per period.

Based on the previous study, we accounted for the vaccine’s time-lag effects, which took at least 40 days to impact the pandemic, and the stringency index of NPIs requires at least 14 days to have their effects [27].

**Figure 1:**
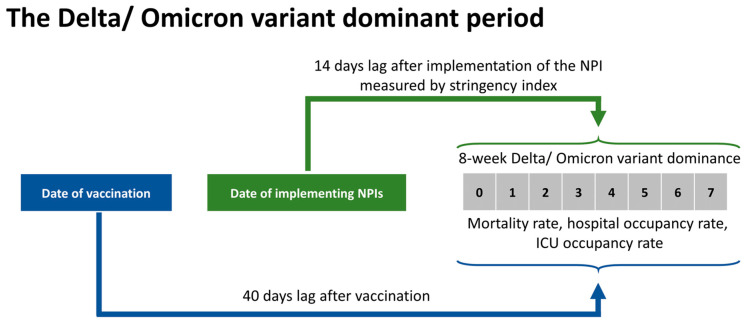
Time lag effects of vaccination and NPIs during study periods.

Figure 1 shows how time lag effects were defined during the variant dominance period.

### 2.4. Statistical Analysis

- Analysis of aggregate data of the 8-week Omicron period and the 8-week Delta period.

We calculated the pandemic indicators over eight weeks in each period. These indicators included total new cases, total new deaths, average daily mortality rate, average daily hospital occupancy rate, average daily ICU occupancy rate, and resilience index. Then, every pandemic indicator would be classified into three equally distributed groups (Tercile 1—Low rate, Tercile 2—Medium rate, Tercile 3—High rate).

Based on the change of resilience index between two periods, we grouped 29 countries into three groups equally: Good performance (Tercile 1): Group of countries with resilience index increased at a low level; Medium performance (Tercile 2): Group of countries with resilience index increased at a medium level; Poor performance (Tercile 3): Group of countries with resilience index increased to a high level. One Way ANOVA was employed to test the difference of country characteristics among the three groups.

- Longitudinal analysis

To explore factors explaining the variation of resilience in each period, we used the linear mixed-effects model with a log transformation of resilience index as our outcome variable was highly skewed. The independent variables included the COVID-19 interventions (vaccine coverage, stringency index of NPIs), government indicators, sociodemographic features, health care capacity, burden of non-communicable diseases, burden of health behavior and environment risks. These factors will be grouped into terciles (three equal groups).

The random intercepts and slopes models were employed to account for the time-varying characteristics of the individual country. We used the forward selection approach based on the Bayesian information criterion (BIC) to find the most optimal model. Firstly, the independent variables showing significance in univariate analysis were selected and ranked in decreasing order based on BIC. Then, we entered these variables sequentially and dropped out those with insignificant statistics.

All analyses were implemented using R programing software (version 4.1.3, R Development Core Team, Vienna, Austria). We utilized the LME4 package for developing the linear mixed effect model, and the performance package to extract the model’s AIC, BIC and R square [28].

## 3. Results

### Characteristics of Selected Countries

Table 1 presents the demographics of selected countries. The median of the rule of law index, the regulatory quality index, and the government effectiveness index were 1.4 (25–75th percentile, 0.9–1.7), 1.2 (25–75th percentile, 0.8–1.6), and 1.3 (25–75th percentile, 1.0–1.6) respectively.

The population median was 10,160,159 (25–75th percentile, 5,453,600–32,776,195), and the population density was 112.371 (25–75th percentile, 65.18, 231.447). The GDP per capita was 38,605.7 (25–75th percentile, 30,155.2–46,682.5).

In terms of health care capacity, the median of the UHC index was 82 (25–75th percentile, 76–84), and the median of the GHS index was 59.3 (25–75th percentile, 54.4–64.7). The median of nurses and physicians per 1000 inhabitants were 3.7 (25–75th percentile, 3.0–4.1) and 10.3 (25–75th percentile, 7.4–12.4), respectively. The median percentage of GDP per capita was 8.7 (25–75th percentile, 7.0–10.2).

The COVID-19 victims with chronic diseases, cancers and cardiovascular diseases had the highest death rate per 100,000 inhabitants with a median of 137.4 (25–75th percentile, 124.2–145.9) and 132.4 (25–75th percentile, 108.1–232.2), respectively, whereas the death rate from diabetes was the lowest with a median of 8.5 (25–75th percentile, 6.4–12.4).

Regarding environmental and health behavior factors, tobacco ranked first in terms of years lived with disability (YLDs) per 100,000 with a median of 536.948 (25–75th percentile, 470.5–595.9). In contrast, zinc deficiency had the lowest YLDs with a median of 0.04 (25–75th percentile, 0.03–0.06).

Table 2 summarizes the stringency of NPIs and levels of vaccine coverage among 29 countries between two periods. The stringency index tended to be stable at around 45–48 points between Delta and Omicron variant periods. Over the Omicron period, the median percentage of people fully vaccinated increased by over 20%, whereas the figure for the percentage of people vaccinated with at least one dose was about 15% compared to the Delta period. At the end of the study period, the median population who received the booster dosage was more than 40%.

**Figure 2:**
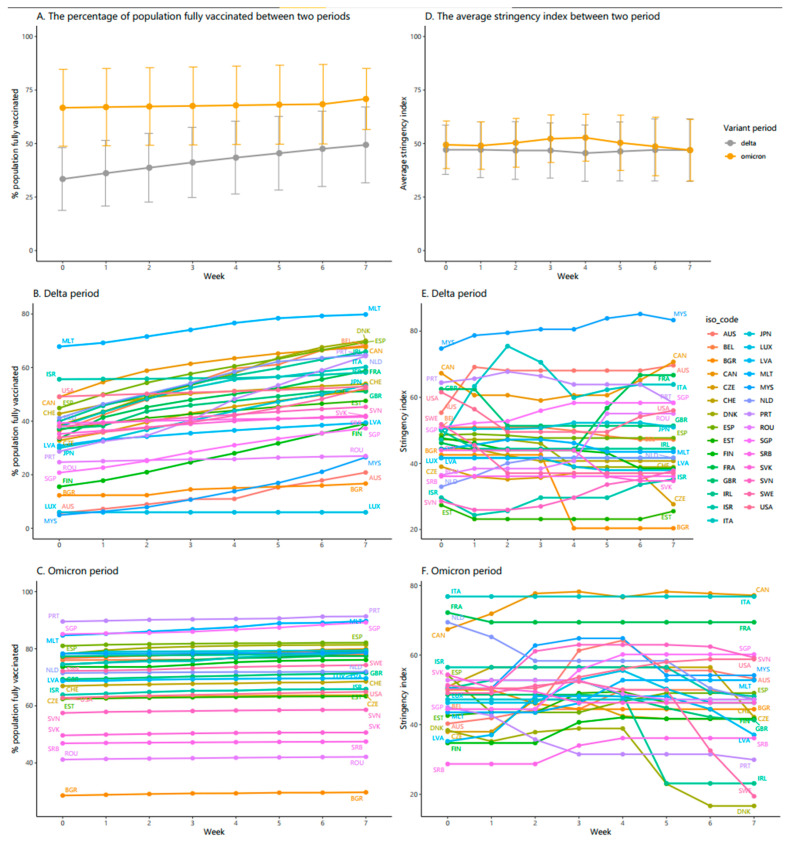
Government response and vaccination coverage across 29 countries over the first 8 weeks of the Delta and Omicron variant periods. Country Abbreviations: AUS—Australia, BEL—Belgium, BGR—Bulgaria, CAN—Canada, CZE—Czechia, CHE—Switzerland, DNK—Denmark, ESP—Spain, EST—Estonia, FIN—Finland, FRA—France, GBR—United Kingdom, IRL—Ireland, ISR—Israel, ITA—Italy, JPN—Japan, LUX—Luxembourg, LVA—Latvia, MLT—Malta, MYS—Malaysia, NLD—Netherlands, PRT—Portugal, ROU—Romania, SGP—Singapore, SRB—Serbia, SVK—Slovakia, SVN—Slovenia, SWE—Sweden, USA—United States.

According to Figure 2A, among 29 countries, the proportion of people fully vaccinated during the Delta variant period followed an increasing trend from week 1 to week 8, by approximately 17%. By contrast, the figure for the Omicron period remained stable between week 1 and week 7 and then witnessed an upward trend to nearly 70% at the end of the period.

Figure 2B presents vaccination during the Delta period, Malta obtained the highest proportion of people with two-dose vaccination, starting at around 68% at week 1 and reaching roughly 80% at week 8. The countries having the percentage of the population fully vaccinated increasing over 60% were Denmark, Spain, Belgium, Ireland, and the Netherlands. The countries where the proportion of people fully vaccinated was dramatically lower were Luxembourg, Bulgaria, and Australia, at just under 20%.

Figure 2C demonstrates over the Omicron period, the proportion of two-dose vaccinations was the highest in Portugal (90%), Malta (88%), and Singapore (87%). However, most countries had two-dose vaccinations ranging from 60% to 80%. Considerably, Bulgaria was the nation with the lowest proportion of people fully vaccinated, at just under 20%.

Figure 2D presents the changes in the average stringency index over the study periods of Delta and Omicron variants. The average stringency index remained stable over the first three weeks at 48 points, slightly decreasing to 46 points and gradually rising afterward during the Delta period. In comparison, the Omicron period witnessed an increase in average stringency index during the first four weeks, peaking at 52 points in week 4 and then dropping gradually over the last four weeks, standing at the same 46 points compared to the Delta period at the end of the study period.

The top panel in Figure 2E indicates that during the Delta period, Malaysia observed the largest increase in the stringency index during the first seven weeks of the study period, reaching a peak of around 82 points at week 7, and declining to 81 points at week 8. The countries that experienced a steep fluctuation and kept the stringency index over 60 points at the end of the study period were Canada, Australia, France, and Italy. It was noticeable that Bulgaria reached the lowest point and remained unchanged in the stringency index during the last four weeks, at just above 20 points.

From the bottom panel in Figure 2F, we can see that throughout the Omicron period, some countries, namely France, Canada, and Italy, kept the highest stringency index, ranging from 70 points to 80 points between week 2 and week 8 of the study periods. Meanwhile, Singapore and Slovenia witnessed an upward trend in stringency index during the first four weeks which slightly declined afterward to under 60 points at week 8. Denmark, Sweden, and Ireland countries underwent a comparable trend over the first four and five weeks; however, they dropped sharply afterward to a stringency index under 25 points.

**Figure 3:**
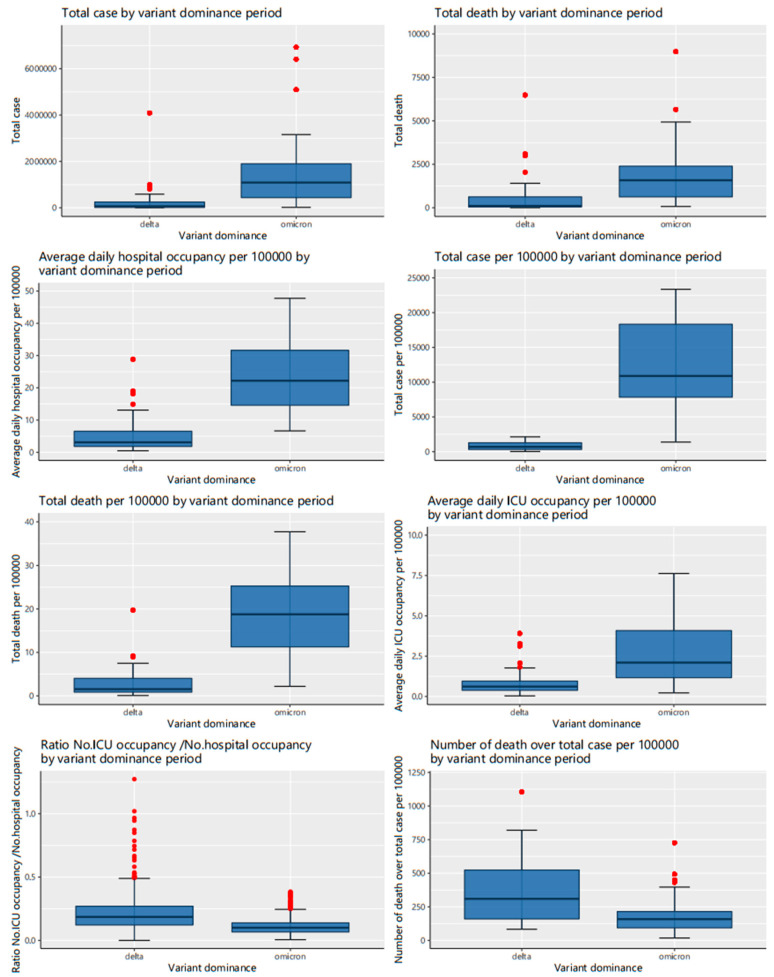
COVID-19 infection characteristics between the Delta and Omicron variant periods.

Figure 3 shows the median of total cases (Delta: 64,169 vs. Omicron: 1,149,543), total deaths (Delta: 160 vs. Omicron: 1600), total cases per 100,000 (Delta: 698.48 vs. Omicron: 11,314.22), total deaths per 100,000 (Delta: 1.53 vs. Omicron: 18.78), the average daily hospital occupancy per 100,000 (Delta: 3.10 vs. Omicron: 24.59), and average daily ICU occupancy per 100,000 (Delta: 0.62 vs. Omicron: 2.10) were found to be considerably higher for Omicron than for Delta. Meanwhile, the ratio of ICU occupancy/hospital occupancy and the number of deaths over infection cases per 100,000 were 0.19 and 316.80, respectively, during the Delta period, approximately doubling the figures for the Omicron period (0.10 and 158.54).

Table 3 displays that throughout the periods of Delta and Omicron variants, the countries with the higher hospitalization rates, higher ICU occupancy rates, higher mortality rates, included Bulgaria (0.637; 0.682; 0.677 respectively), Latvia (0.506; 0.445; 0.437 respectively), and Slovakia (0.415; 0.459; 0.394 respectively). Slovenia observed the most significant increase in ICU occupancy rate and the third-largest rise in mortality rate, with 0.7 and 0.399, respectively. Meanwhile, Malaysia witnessed the largest decreases in three indicators: hospitalization, ICU, and mortality; the figures accounted for −0.214; −0.363, and −0.205, respectively. Likewise, Japan experienced the second-largest decline in hospital and ICU occupancy rates, reaching −0.028 and −0.106, respectively, whereas the mortality rate slightly decreased by 0.023. The Netherlands and Singapore experienced significant decreases in all three indicators: hospital, ICU, and mortality. Overall, the top five countries with the largest positive shifts in the resilience index were Malaysia (−0.26), Japan (−0.037), Singapore (0.041), the Netherlands (0.055) and Finland (0.111).

**Figure 4:**
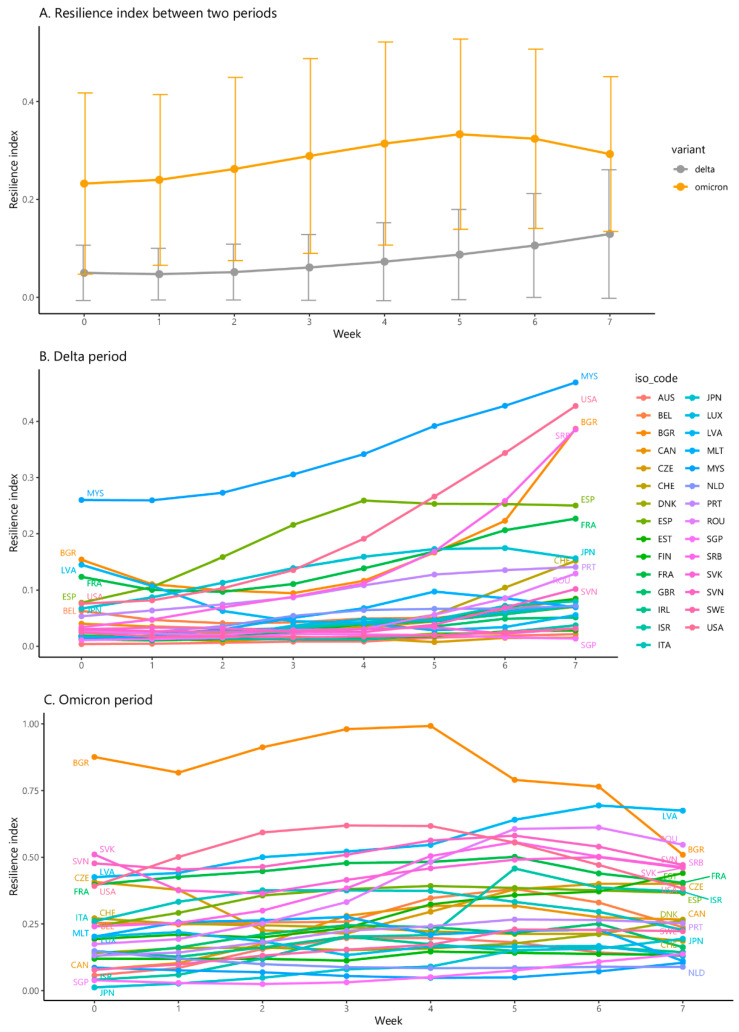
The evolution of the resilience index across 29 countries over the first 8 weeks during the Delta and Omicron variant periods. Country Abbreviations: AUS—Australia, BEL—Belgium, BGR—Bulgaria, CAN—Canada, CZE—Czechia, CHE—Switzerland, DNK—Denmark, ESP—Spain, EST—Estonia, FIN—Finland, FRA—France, GBR—United Kingdom, IRL—Ireland, ISR—Israel, ITA—Italy, JPN—Japan, LUX—Luxembourg, LVA—Latvia, MLT—Malta, MYS—Malaysia, NLD—Netherlands, PRT—Portugal, ROU—Romania, SGP—Singapore, SRB—Serbia, SVK—Slovakia, SVN—Slovenia, SWE—Sweden, USA—United States.

Figure 4A indicates that the Omicron period underwent poorer resilience index evolution than the Delta period. The Delta period experienced a gradually increasing trend in terms of a resilience index during the first eight weeks. Similarly, the Omicron period witnessed an upward trend until the 6th week and followed a decreasing trend afterward.

Figure 4B shows the evolution of the resilience index varied dramatically across countries during the first eight weeks of the Delta variant period. The resilience index among countries, namely Malaysia and the United States, had become serious, ranging from 0.26 to 0.469 and 0.076 to 0.427, respectively. The countries where the resilience index rose in the first 5–6 weeks and followed a decreasing trend in the final week of the period, were Japan Malta, and Spain, whereas some countries, namely Israel, Sweden, and Czech maintained a slightly steady resilience index during the whole period.

Figure 4C demonstrates that the progression of the resilience index varied dramatically across countries during the first eight weeks of the Omicron variant period. Most countries observing a resilience index that increased from week 1 to week 4 and week 5, and declined afterward were Australia, Bulgaria, Spain, United States. Meanwhile, countries where the resilience index kept rising considerably during the period, include Czechia, Denmark and Latvia.

**Figure 5:**
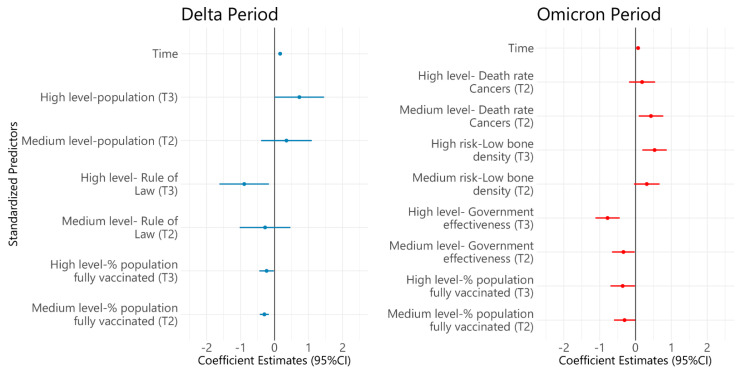
Multivariate linear mixed effect models of factors associated with the resilience index.

The results of the multivariate linear mixed-effects model to explore the factors associated with the resilience index in each period are shown in Figure 5. During the delta variant dominance period, country population (T3 vs. T1: β = 0.73, 95%CI: 0.003–1.456), was positively associated with the log transformation of the resilience index, whereas the rule of law index (T3 vs. T1: β = −0.892, 95%CI: (−1.62)–(−0.164)), the percentage of people fully vaccinated (T3 vs. T1: β = −0.233, 95%CI: −0.447–(−0.019)) had negative associations. In the time of Omicron variant dominance, the factors positively associated with the log transformation of the resilience index were the rate of YLD caused by low bone density (T3 vs. T1: β = 0.531, 95%CI: 0.191–0.871) and the death rate caused by cancers (T3 vs. T1: β = 0.43, 95%CI: 0.088–0.773). In contrast, the percentage of the population fully vaccinated (T3 vs. T1: β = −0.358, 95%CI: (−0.7)–(−0.017)) and the government effectiveness index were significantly associated with the lower log transformation of the residence index (T3 vs. T1: β = −0.78, 95%CI: (−1.118)–(−0.443)).

Based on the level of increase in resilience index between the Delta and Omicron periods, some characteristics of countries with a positive change in resilience index (good performance) were defined in Table 4. Compared to the poor performance group, the countries with good performance in the resilience index have the lower stringency index, higher vaccination coverage rate (one dose and fully vaccinated), and higher government indicators (the rule of law, regulatory quality, government effectiveness). In terms of socio-economic characteristics, the significantly higher score of life expectancy, GDP per capita, and the percentage of the population living in urban areas were observed in the countries with good performance. The good performance group had a better UHC index score regarding health capability. In addition, a lower death rate of non-communicable diseases, cancers, and cardiovascular diseases was observed in the good performance group. These countries with good performance also have less YLD caused by Vitamin A deficiency, tobacco, and PM2.5 than those with poor performance.

## 4. Discussion

From our literature review, this study is the first nation-wide comprehensive exploration to provide the latest evidence on the COVID-19 resilience index during the Delta and Omicron variant periods. Unlike other research reports that mostly focused only on a specific COVID-19 indicator (e.g., incidence, mortality rate, and death), this study investigated the resilience index, which is a combination of mortality rate, hospital occupancy rate, and ICU occupancy rate. The index supports governments in keeping track of the COVID-19 situation and measuring its burden on the health care system more accurately than the COVID-19 incidence. Besides, the comparison of the resilience index over the first eight weeks during the Omicron and Delta variant periods provides a better understanding of how governments coped with the new variants and explores possible factors associated with government performance.

With lowered death cases over total cases and the ratio of the number of ICU occupancies and hospital occupancies during the first eight weeks of each period, the Omicron variant period was more likely to be less severe than the Delta period. This finding is supported by previous studies in South Africa [5] and the United States [29]. However, our data also indicated that the overall burden from the Omicron variant was much higher than that of the Delta variant due to the considerable escalation in total cases, total deaths, hospital occupancy rate, and ICU occupancy rate (Figure 3). Previous research has shown that the Omicron variant has a higher transmission ability [6,17] and increases the number of vaccine breakthrough cases [29]. Despite the lesser severity, the Omicron variant still creates a huge burden on the health care system and society. Therefore, it is necessary to raise the community’s awareness of the severity of Omicron, and governments need to keep track of COVID-19 indicators and new variants closely. We found that Bulgaria, Slovakia, and Slovenia witnessed the highest burden caused by Omicron, and they all had a common characteristic which was a low percentage of the population fully vaccinated. This suggests these governments should establish more policies or solutions to improve the vaccination rate across the population.

In this study, we found factors associated with the resilience index and identified characteristics of countries with good performance during the Delta and Omicron periods.

Full vaccine coverage and government indicators were strongly and consistently associated with a lower resilience index in the Delta and Omicron periods. In the group of countries with good performance in the change of the resilience index over the two periods, the median full vaccination rate reached nearly 80% (78.9, 25th–75th: 74.7, 79.4), whereas this figure for the poor performance group was around 60% (61.2, 25th–75th: 48.3, 68.5). Hence, reducing vaccine hesitancy, and barriers to vaccine access [30] are key tasks to improve the vaccine coverage rate. A study published in Lancet pointed out that trust in government and interpersonal trust might be significant contributors to vaccine acceptance [31]. Therefore, further studies on factors influencing the vaccination rate in these countries are necessary.

The government indicators (government effectiveness and the rule of law) were significantly associated with a better resilience index in each variant dominance period and were higher in counties with good performance. Our finding is supported by previous studies [8,15]. These government indices measure the ability of the government to establish and implement regulations and the government’s credibility to the community [22]. Since COVID-19 has created a large and profound impact on every sector of society, good governance has become more important than ever to integrate different resources in the battle against the COVID-19 pandemic. Previous studies have found that government trust might be associated with compliance with public health measures and vaccine hesitancy [15,31]. Therefore, governments need to strengthen the socio-economic components and increase their credibility among communities to facilitate effective and synchronized policy responses [32].

Interestingly, results indicated that the high-level intensity of NPIs did not show a significant decrease in controlling the pandemic. In Table 4, we found the inverse trend, the countries with poor performance had a higher stringency level. This is appropriate since most countries reach high-levels of vaccination; decreasing the level of stringency to recover the economy is an imminent need [11,30]. Otherwise, countries with a high mortality rate, hospitalization occupancy, and ICU occupancy are more likely to recirculate NPIs to reduce the burden on the health care system.

The results found that the countries with higher performance have a higher life expectancy, GDP per capita, population living in urban areas, and UHC indexes. However, we did not find any factors related to health capacity and socio-economic characteristics that significantly associated with the resilience index during the Delta or Omicron periods. Because socio-economic status is highly correlated with governance indicators and vaccine coverage, they should be interpreted cautiously. Further studies investigating a larger number of countries, and a longer period is needed to explore these associations.

In terms of global health burden, we found that factors associated with the resilience index in the Omicron period are death rates caused by cancers. When we compared these health burden factors among the performance groups, the results showed that the poor performance group has a higher death rate from non-communicable diseases and specific causes, including cardiovascular diseases and cancers. Previous studies indicated a similar finding that COVID-19 patients with chronic conditions have a higher risk of ICU admission and mortality [33]. Therefore, patients with chronic diseases should be monitored thoroughly by healthcare professionals. Policymakers need to develop programs to protect the vulnerable COVID-19 patient groups.

The countries with a higher health burden of low bone density were more likely to have poorer resilience during the Omicron period; and the good performance group witnessed the lower YLD caused by vitamin A deficiency when compared to the poor performance group over the two periods. Hence, nutritional factors might play a key role in explaining the variation in the resilience index, particularly low bone density. Our finding is in line with the previous studies. As the low bone density condition may reflect the risk of vitamin D deficiency, many studies show that hypovitaminosis D is highly associated with a higher risk of respiratory infections, admissions to the intensive care unit, and mortality among COVID-19 patients [34,35]. Similarly, other studies showed that a low level of vitamin A in plasma is significantly linked with increased inflammatory markers (CRP, ferritin) that lead to a higher risk of mortality among COVID-19 hospitalized patients [36,37]. Therefore, governments and public health professionals need to conduct more evaluations on the role of nutrition conditions on COVID-19 patients in preventing severity and reducing COVID-19 mortality.

We also found that the good performance countries had a lower YLD of PM2.5. The previous study showed that places with high PM2.5 were more likely to have a high mortality rate [38]. Most studies, however, were limited to establishing a correlation or association between PM concentration and the COVID-19 situation. As a result, further research on different study designs would be required to establish a scientific causal relationship between PM2.5 and COVID-19 severity.

Our study admits several limitations. Firstly, our data were obtained from 29 countries, which does not represent the global scenario, particularly those in low and middle-income countries where hospitalization and ICU admission statistics are limited or missing. And these financially challenged countries are those truly in need of guidance and help from our investigation. Furthermore, because the data in our study was primarily collected at the aggregated national level, data from the regional or state level were not included in our analysis. A further examination of regional data would be more useful and informative when these data become available. Second, because our data were mostly based on government-reported statistics, the bias of underreported and missed detection are inevitable. We calculated the weekly average of the hospitalization occupancy rate, ICU occupancy rate, death rate, and stringency index to overcome this constraint. Third, our study might not have included all the major factors possibly associated with the COVID-19 cases, such as meteorological factors, vaccine hesitancy, pandemic fatigue, and level of public trust during COVID-19. Additional works are needed to gain greater understanding when these data become accessible. Finally, because the association of contextual variables in our ecological study (e.g., chronic disease burden, environmental and health behavior risk) might be influenced by socio-demographic factors and differ from the association observed at the individual level, our findings should be interpreted with caution. Our findings should be served as reference points for further individual-level studies.

## 5. Conclusions

The results among 29 countries showed that the Omicron variant causes a lower rate of mortality among COVID-19 patients; however, it is still imposing a huge burden on the health care system and society in terms of total death, high hospital and ICU occupancy rate. Therefore, it is necessary to raise the community’s awareness of publications on the severity of Omicron, and the government needs to keep track of COVID-19 indicators and new variants closely. This study found that the percentage of the population fully vaccinated and high government indices were significantly associated with a better resilience index. In contrast, the higher death rate of cancers and greater years lived with disability (YLD) caused by low bone density were linked with a poor resilience index in the Omicron periods. Generally, over two periods of Delta and Omicron, countries with good performance in pandemic control have higher vaccination coverage rates, government indices, GDP per capita, % population living in urban areas, lower death rates from chronic diseases, and lower YLD caused by nutritional deficiency and PM2.5. Based on our findings, we suggest that it is necessary to raise the community’s awareness with the utmost importance on the severity of Omicron. The government needs to keep enhancing the vaccine coverage rates and develop interventions for chronic diseases and nutritional deficiency to mitigate the stark COVID-19 burden.

## Figures and Tables

**Table 1:** The demographics of selected countries.

Characteristic	Mean (SD)	Median (25th Percentile, 75th Percentile)
Government indicators		
Rule of law	1.2 (0.6)	1.4 (0.9, 1.7)
Regulatory quality	1.3 (0.5)	1.2 (0.8, 1.6)
Government effectiveness	1.2 (0.6)	1.3 (1.0, 1.6)
Socioeconomic characteristics		
Population	32,509,300.6 (64,315,654.6)	10,160,159 (5,453,600, 32,776,195)
Population density	458.3 (1460.4)	112.371 (65.2, 231.4)
Life expectancy	80.9 (2.9)	82.1 (78.9, 82.8)
GDP per capita (USD per capita)	40,988.8 (17,790.5)	38,605.7 (30,155.2, 46,682.5)
% Population aged over 65	18.0 (3.9)	18.8 (15.5, 19.7)
% Population living in urban area	78.3 (13.5)	80.7 (69.1, 88.0)
Health care capacity		
UHC index	79.9 (6.1)	82 (76, 84)
No. physicians per 1000	3.7 (0.9)	3.7 (3.0, 4.1)
No. nurses and midwives per 1000	10.4 (3.9)	10.3 (7.4, 12.4)
% GDP for health expenditure	8.7 (2.6)	8.672 (7.0, 10.2)
GHS index	58.8 (8.7)	59.3 (54.4, 64.7)
Health burden of chronic diseases (death rate per 100,000)		
Non-communicable diseases	431.8 (139.1)	383.7 (347.6, 484.6)
Diabetes	9.8 (5.1)	8.5 (6.4, 12.7)
Chronic respiratory diseases	21.2 (8.6)	19.9 (14.6, 27.6)
Cancers	135.1 (18.9)	137.4 (124.2, 145.9)
Chronic kidney diseases	10.5 (4.8)	9.7 (8.0, 11.1)
Cardiovascular diseases	183.6 (117.8)	132.4 (108.1, 232.2)
Health burden of environmental and health behavior risk (Years lived with disability (YLDs) per 100,000)		
PM2.5	84.3 (57.3)	69.0 (45.5, 118.3)
Tobacco	551.6 (117.6)	536.9 (470.5, 595.9)
Zinc deficiency	0.05 (0.04)	0.04 (0.03, 0.06)
Vitamin A deficiency	1.1 (1.9)	0.3 (0.2, 0.6)
Low bone density	135.3 (35.2)	137.0 (110.3, 149.2)

**Table 2:** Vaccine and stringency index characteristics between two periods.

Characteristic	Delta		Omicron	
	Mean (SD)	Median (Q1, Q3)	Mean (SD)	Median (Q1, Q3)
Stringency index	46.7 (12.6)	45.1 (39.9, 53.3)	50.0 (11.0)	48.2 (44.2, 54.5)
% Population vaccinated at least one dose	59.4 (15.7)	62.9 (49.8, 72.3)	73.5 (16.3)	78.3 (69.71, 83.3)
% Population fully vaccinated	49.4 (17.7)	52.7 (39.5, 64.3)	70.9 (14.3)	74.3 (64.9, 79.2)
% Population vaccinated the booster dose	0.3 (1.1)	0 (0, 0.01)	41.9 (18.8)	43.8 (29.9, 55.8)

**Table 3:** The average of standardized values of hospital occupancy rate, ICU occupancy rate, mortality rate, and resilience index across countries between the two periods.

Country	Average Daily Hospital Occupancy per 100,000 Inhabitants (aHOSP) ^a^			Average Daily ICU Occupancy per 100,000 Inhabitants (aICU) ^a^			Average Daily Mortality Rate per 100,000 Inhabitants (aMOR) ^a^			Average Resilience Index (aRESIDX)		
	Delta	Omicron	Change aHOSP (Rank) ^b^	Delta	Omicron	Change aICU (Rank) ^c^	Delta	Omicron	Change aMOR (Rank) ^d^	Delta	Omicron	Change aRESIDX (Rank) ^e^
Australia	0.006	0.152	0.146 (11)	0.012	0.129	0.117 (11)	0.012	0.141	0.129 (6)	0.01	0.141	0.131 (8)
Belgium	0.031	0.282	0.251 (18)	0.106	0.389	0.283 (21)	0.028	0.21	0.182 (13)	0.055	0.294	0.239 (18)
Bulgaria	0.187	0.824	0.637 (29)	0.189	0.871	0.682 (28)	0.113	0.79	0.677 (29)	0.163	0.828	0.665 (29)
Canada	0.018	0.191	0.173 (14)	0.081	0.256	0.175 (16)	0.025	0.195	0.17 (11)	0.041	0.214	0.173 (15)
Czechia	0.004	0.295	0.291 (21)	0.014	0.4	0.386 (25)	0.022	0.303	0.281 (21)	0.013	0.333	0.32 (23)
Denmark	0.012	0.173	0.161 (12)	0.023	0.1	0.077 (7)	0.019	0.239	0.22 (18)	0.018	0.171	0.153 (12)
Estonia	0.039	0.366	0.327 (24)	0.044	0.164	0.12 (12)	0.026	0.316	0.29 (22)	0.036	0.282	0.246 (19)
Finland	0.009	0.125	0.116 (6)	0.02	0.095	0.075 (6)	0.021	0.163	0.142 (9)	0.017	0.128	0.111 (5)
France	0.134	0.452	0.318 (22)	0.221	0.592	0.371 (23)	0.064	0.28	0.216 (17)	0.14	0.441	0.301 (22)
Ireland	0.026	0.158	0.132 (8)	0.055	0.191	0.136 (15)	0.032	0.127	0.095 (5)	0.038	0.159	0.121 (7)
Israel	0.015	0.259	0.244 (17)	0.023	0.218	0.195 (18)	0.018	0.216	0.198 (15)	0.019	0.231	0.212 (17)
Italy	0.042	0.332	0.29 (20)	0.049	0.264	0.215 (19)	0.039	0.367	0.328 (23)	0.043	0.321	0.278 (21)
Japan	0.159	0.131	−0.028 (2)	0.196	0.09	−0.106 (2)	0.03	0.053	0.023 (2)	0.128	0.091	−0.037 (2)
Latvia	0.036	0.542	0.506 (28)	0.115	0.56	0.445 (26)	0.057	0.494	0.437 (28)	0.069	0.532	0.463 (27)
Luxembourg	0.022	0.117	0.095 (5)	0.058	0.238	0.18 (17)	0.027	0.164	0.137 (8)	0.036	0.173	0.137 (10)
Malaysia	0.305	0.091	−0.214 (1)	0.431	0.068	−0.363 (1)	0.255	0.05	−0.205 (1)	0.33	0.07	−0.26 (1)
Malta	0.052	0.194	0.142 (9)	0.043	0.11	0.067 (5)	0.055	0.32	0.265 (20)	0.05	0.208	0.158 (13)
Netherlands	0.021	0.072	0.051 (3)	0.1	0.183	0.083 (9)	0.028	0.061	0.033 (4)	0.05	0.105	0.055 (4)
Portugal	0.068	0.197	0.129 (7)	0.155	0.172	0.017 (3)	0.058	0.245	0.187 (14)	0.094	0.205	0.111 (6)
Romania	0.049	0.449	0.4 (26)	0.049	0.401	0.352 (22)	0.045	0.299	0.254 (19)	0.048	0.383	0.335 (25)
Serbia	0.178	0.519	0.341 (25)	0.084	0.207	0.123 (13)	0.113	0.451	0.338 (24)	0.125	0.392	0.267 (20)
Singapore	0.033	0.111	0.078 (4)	0.004	0.025	0.021 (4)	0.012	0.035	0.023 (3)	0.016	0.057	0.041 (3)
Slovakia	0.015	0.43	0.415 (27)	0.058	0.517	0.459 (27)	0.018	0.412	0.394 (26)	0.03	0.453	0.423 (26)
Slovenia	0.026	0.348	0.322 (23)	0.061	0.761	0.7 (29)	0.029	0.428	0.399 (27)	0.039	0.512	0.473 (28)
Spain	0.139	0.303	0.164 (13)	0.336	0.46	0.124 (14)	0.088	0.24	0.152 (10)	0.188	0.334	0.146 (11)
Sweden	0.016	0.158	0.142 (10)	0.029	0.115	0.086 (10)	0.017	0.193	0.176 (12)	0.021	0.155	0.134 (9)
Switzerland	0.032	0.231	0.199 (15)	0.087	0.319	0.232 (20)	0.023	0.156	0.133 (7)	0.047	0.235	0.188 (16)
United Kingdom	0.024	0.24	0.216 (16)	0.039	0.117	0.078 (8)	0.027	0.241	0.214 (16)	0.03	0.199	0.169 (14)
United States	0.123	0.379	0.256 (19)	0.33	0.711	0.381 (24)	0.098	0.458	0.36 (25)	0.184	0.516	0.332 (24)

Green: Tercile 1—Group of countries with change values increased to a low level; Grey: Tercile 2—Group of countries with change values increased to a medium level; Orange: Tercile 3—Group of countries with change values increased to a high level. ^a^ Values were standardized into a range from 0 to 1; ^b^ The change in the average of standardized daily hospital occupancy per 100,000 inhabitants (aHOSP Change) = aHOSP of the Omicron period—aHOSP of the Delta period. Ranking in decreasing order—First place for a country with the lowest aHOSP Change. ^c^ The change in the average of the standardized daily ICU occupancy per 100,000 inhabitants (aICU Change) = aICU of the Omicron period—aICU of the Delta period. Ranking in decreasing order—First place for a country with the lowest aICU change. ^d^ The change in the average of the standardized daily mortality rate per 100,000 inhabitants (aMOR Change) = aMOR of the Omicron period—aMOR of the Delta period. Ranking in decreasing order—First place for a country with the lowest aMOR Change. ^e^ The change in the average of standardized stringency index (aRESIDX Change) = Standardized average stringency index of the Omicron period—Standardized average stringency index of the Delta period. Ranking in decreasing order—First place for a country with the lowest aRESIDX Change.

**Table 4:** Characteristics of countries having worse, medium and better performance in terms of COVID-19 resilience index.

Factors	Country Performance ^a^	Mean (SD)	Median (Q1,Q3)	*p* Value
Difference in intensity of stringency ^b^	Worse	9.3 (9.5)	7.1 (2.2, 14.2)	0.040
	Medium	5.8 (12.8)	3.8 (−2, 12.4)	
	Good	−5.2 (14.6)	−4 (−12, 2.7)	
% Population vaccinated booster dose ^c^	Worse	30.5 (18)	29.1 (27.1, 35.6)	0.032
	Medium	52.2 (10.8)	55.4 (43.8, 61.5)	
	Good	44.1 (20.4)	50.9 (42, 56.8)	
% Population fully vaccinated ^d^	Worse	58.3 (15.8)	61.2 (48.3, 68.5)	0.001
	Medium	75.7 (8.8)	78.1 (68.5, 81.4)	
	Good	79.1 (6.6)	78.9 (74.7, 79.4)	
% Population vaccinated at least one dose ^e^	Worse	59.7 (19.7)	62.8 (49.4, 75.2)	0.001
	Medium	78.9 (8.8)	79.3 (72.1, 85.3)	
	Good	82.5 (6.2)	80.9 (78.8, 84.2)	
Government indicators				
Rule of Law	Worse	0.7 (0.6)	0.8 (0.3, 1.1)	0.000
	Medium	1.4 (0.4)	1.4 (1, 1.7)	
	Good	1.6 (0.4)	1.7 (1.5, 1.8)	
Regulatory Quality	Worse	0.8 (0.4)	0.9 (0.5, 1.2)	0.001
	Medium	1.4 (0.3)	1.5 (1.2, 1.6)	
	Good	1.6 (0.5)	1.7 (1.4, 1.8)	
Government Effectiveness	Worse	0.6 (0.6)	0.7 (0.1, 1.1)	0.000
	Medium	1.4 (0.4)	1.3 (1.1, 1.6)	
	Good	1.6 (0.4)	1.7 (1.5, 1.9)	
Socio-economic characteristics				
Life expectancy	Worse	78.6 (3.1)	78.2 (76, 80.8)	0.004
	Medium	82 (1.6)	82.4 (81.3, 83)	
	Good	82.1 (2.3)	82.3 (82.1, 83.3)	
GDP per capita	Worse	30,320.2 (11,305.1)	30,778 (23,750.9, 34,566.5)	0.017
	Medium	40,435.7 (8456.5)	39,753.2 (34,272.4, 44,017.6)	
	Good	52,155.2 (22,977.9)	45,799 (39,398.1, 62,619.6)	
% Population aged over 65	Worse	18.7 (2.4)	19 (17.5, 19.7)	0.679
	Medium	18 (2.5)	18.6 (18.4, 19.4)	
	Good	17.2 (5.8)	17.1 (14, 20.9)	
% Population living in Urban area	Worse	67 (11.4)	69.5 (55.2, 75)	0.002
	Medium	84.6 (9.6)	83.7 (80.6, 92.5)	
	Good	84 (11.8)	86.9 (78.8, 91.6)	
Population density	Worse	97.9 (51.6)	93.9 (69, 120.2)	0.436
	Medium	331.6 (443.9)	214.2 (93.1, 375.6)	
	Good	932.8 (2459)	104.3 (36, 318.7)	
Population	Worse	51,371,999.9 (101,786,678.4)	8,810,604 (5,804,839.3, 50,057,546.3)	0.533
	Medium	21,145,961.8 (23,967,521.1)	9,291,000 (5,813,302, 38,067,913)	
	Good	23,873,606.3 (37,291,513.1)	10,164,041 (5,477,290.3, 23,634,436.3)	
Health care capacity				
UHC index	Worse	75.2 (6.3)	76.5 (71.8, 78.8)	0.005
	Medium	82.9 (3.9)	83 (82, 84)	
	Good	82 (4.8)	83 (79, 86)	
GHS index	Worse	57.7 (9.7)	57.2 (52.1, 61.9)	0.768
	Medium	58.1 (9.5)	59.3 (55.5, 64.4)	
	Good	60.4 (7.4)	59 (55.6, 64.9)	
No. physicians per 1000	Worse	3.8 (1)	3.5 (3.1, 4.1)	0.542
	Medium	3.8 (0.7)	4 (3.7, 4.3)	
	Good	3.4 (1.1)	3.6 (2.6, 4.1)	
No. nurses and midwives per 1000	Worse	8.6 (2.7)	7.5 (7.1, 9.6)	0.156
	Medium	12 (4.4)	10.4 (9.5, 14.5)	
	Good	10.9 (4)	12 (8, 12.5)	
% GDP for health expenditure	Worse	8.8 (3.2)	8.2 (7, 8.7)	0.538
	Medium	9.4 (1.6)	10 (8.2, 10.7)	
	Good	8 (2.8)	9.3 (5.7, 10.1)	
Health burden of chronic diseases				
Death rate of NCD per 100,000	Worse	542.1 (170.8)	521.2 (408.9, 634.9)	0.004
	Medium	382.5 (63.2)	356.6 (347.6, 409.9)	
	Good	365.8 (81.7)	364.6 (342.9, 384.9)	
Death rate of diabetes per 100,000	Worse	11.9 (5.2)	10.6 (7.8, 15)	0.204
	Medium	9.6 (5.1)	8.1 (6.4, 11.8)	
	Good	7.8 (4.6)	7.5 (4.5, 9.2)	
Death rate of chronic respiratory disease per 100,000	Worse	18.7 (8.2)	17.6 (12.7, 20.7)	0.506
	Medium	23.4 (9.6)	24.2 (15.4, 29)	
	Good	21.8 (8.4)	21.8 (15.4, 27.5)	
Death rate of cancers per 100,000	Worse	148.7 (15)	145.2 (141.2, 151.7)	0.014
	Medium	133.7 (16.9)	133.3 (124.2, 144.8)	
	Good	125.1 (18)	124.5 (116.4, 130.2)	
Death rate of chronic kidney diseases per 100,000	Worse	9.7 (4.6)	8.2 (6.3, 12.8)	0.818
	Medium	10.8 (4.4)	9.9 (9.5, 11.1)	
	Good	11 (5.6)	10 (8.9, 10.7)	
Death rate of CVD per 100,000	Worse	278.3 (151.3)	265.6 (152.6, 373.9)	0.004
	Medium	133.9 (53.4)	118.6 (107.1, 132.4)	
	Good	133.6 (50.2)	121.9 (109.3, 137.1)	
Heath burden of environmental and health behavior risk				
YLDs caused by PM2.5	Worse	126.9 (65.3)	124.7 (85.8, 169.3)	0.009
	Medium	59.9 (24.9)	59.5 (45.5, 77.6)	
	Good	63.5 (48.1)	56.4 (26.7, 73)	
YLDs caused by tobacco	Worse	626.9 (129.1)	595 (516.5, 751.7)	0.014
	Medium	548.1 (78.2)	536.9 (508.4, 595.9)	
	Good	479.4 (93.8)	468.3 (447.4, 557.4)	
YLDs caused by zinc deficiency	Worse	0.1 (0)	0.1 (0, 0.1)	0.284
	Medium	0 (0)	0 (0, 0.1)	
	Good	0 (0)	0 (0, 0)	
YLDs caused by vitamin A deficiency	Worse	2.5 (2.6)	1.9 (0.3, 3.9)	0.010
	Medium	0.5 (0.8)	0.3 (0.2, 0.4)	
	Good	0.3 (0.2)	0.2 (0.2, 0.3)	
YLDs caused by low bone density	Worse	156.1 (32.5)	147.9 (140.8, 175.7)	0.058
	Medium	128.2 (25.9)	123.9 (113, 133.1)	
	Good	120.9 (38)	120.5 (92.7, 140.7)	

^a^ Country performance was classified into three groups equally based on the level of increase in resilience index between the Delta and Omicron periods (Table 3). Good performance (Tercile 1): Group of countries with resilience index increased to a low level; Medium performance (Tercile 2): Group of countries with resilience index increased to a medium level; Poor performance (Tercile 3): Group of countries with resilience index increased to a high level. ^b^ Difference in intensity of stringency refers to the difference in average intensity of stringency in each country between the Delta period and the Omicron period. ^c^ % population vaccinated with booster dose refers to the percentages of population vaccinated with booster dose at the final week (8th week) of the Omicron period. ^d^ % population fully vaccinated refers to the percentage of population fully vaccinated at the final week (8th week) of the Omicron period. ^e^ % Population vaccinated with at least one dose refers to the percentages of population vaccinated with at least one dose by the final week (8th week) of the Omicron period.

## Data Availability

Data available in a publicly accessible repository. The data presented in this study are available in Supplementary Material. (Table S7. List of variables and data sources).

## Supplementary Materials

The following supporting information can be downloaded at: https://www.mdpi.com/article/10.3390/vaccines10060940/s1, Table S1. Univariate model results and sequence in which covariate were added in the forward selection multivariate linear mixed model during the Delta variant dominant period; Table S2. The final model of multivariate linear mixed effect model in the Delta variant dominant period; Table S3. The variance inflation factor (VIF) for covariates in the mLME during the Delta variant dominant period; Table S4. Univariate model results and sequence in which covariates were added in the forward selection multivariate linear mixed effect model (mLME) during the Omicron variant dominant period; Table S5. The final model of the multivariate linear mixed effect model in the Omicron variant dominant period; Table S6. The variance inflation factor (VIF) for covariates in the mLME during the Omicron variant dominant period; Table S7. List of variables and their data sources; Table S8. The study period of countries during the Delta variant and the Omicron variant periods; Table S9. The variation of stringency index in 29 countries during the first eight weeks of the Delta period; Table S10. The variation of stringency index in 29 countries during the first eight weeks of the Omicron period; Table S11. The percentage of population fully vaccinated in 29 countries during the first eight weeks of the Delta period; Table S12. The percentage of the population fully vaccinated in 29 countries during the first eight weeks of the Omicron period; Table S13. The variation of standardized resilience index in 29 countries during the first eight weeks of the Delta period; Table S14. The variation of standardized resilience index in 29 countries during the first eight weeks of the Omicron period; Table S15. The unstandardized average number of daily hospital occupancy, daily ICU occupancy and mortality rate per 100,000 inhabitants; Figure S1: The residuals across all countries and over the time for the mLME during the Delta variant dominant period; Figure S2: The residuals in the intercepts and slopes across countries and in the between-country intercept and slope residuals for mLME during the Delta variant dominant period; Figure S3: The residuals across all countries and over time for the mLME during the Omicron variant dominant period; Figure S4: The residuals in the intercepts and slopes across countries and in the between-country intercept and slope residuals for mLME during the Omicron variant dominant period.

## References

[1] Patel M.D., Rosenstrom E., Ivy J.S., Mayorga M.E., Keskinocak P., Boyce R.M., Hassmiller Lich K., Smith R.L., Johnson K.T., Delamater P.L. (2021). Association of Simulated COVID-19 Vaccination and Nonpharmaceutical Interventions with Infections, Hospitalizations, and Mortality. JAMA Netw. Open.

[2] Johns Hopkins Coronavirus Resource Center Pandemic Data Initiative. https://coronavirus.jhu.edu/pandemic-data-initiative.

[3] Lin T.-Y., Liao S.-H., Lai C.-C., Paci E., Chuang S.-Y. (2021). Effectiveness of Non-Pharmaceutical Interventions and Vaccine for Containing the Spread of COVID-19: Three Illustrations before and after Vaccination Periods. J. Formos. Med. Assoc..

[4] Jecker N.S., Au D.K.S. (2022). Does Zero-COVID Neglect Health Disparities?. J. Med. Ethics.

[5] Dyer O. (2021). COVID-19: Omicron Is Causing More Infections but Fewer Hospital Admissions than Delta, South African Data Show. BMJ.

[6] Karim S.S.A., Karim Q.A. (2021). Omicron SARS-CoV-2 Variant: A New Chapter in the COVID-19 Pandemic. Lancet.

[7] Andrews N., Stowe J., Kirsebom F., Toffa S., Rickeard T., Gallagher E., Gower C., Kall M., Groves N., O’Connell A.-M. (2022). COVID-19 Vaccine Effectiveness against the Omicron (B.1.1.529) Variant. N. Engl. J. Med..

[8] Coccia M. (2022). Preparedness of Countries to Face COVID-19 Pandemic Crisis: Strategic Positioning and Factors Supporting Effective Strategies of Prevention of Pandemic Threats. Environ. Res..

[9] Bo Y., Guo C., Lin C., Zeng Y., Li H.B., Zhang Y., Hossain M.S., Chan J.W.M., Yeung D.W., Kwok K.O. (2021). Effectiveness of Non-Pharmaceutical Interventions on COVID-19 Transmission in 190 Countries from 23 January to 13 April 2020. Int. J. Infect. Dis..

[10] Haug N., Geyrhofer L., Londei A., Dervic E., Desvars-Larrive A., Loreto V., Pinior B., Thurner S., Klimek P. (2020). Ranking the Effectiveness of Worldwide COVID-19 Government Interventions. Nat. Hum. Behav..

[11] Huy L.D., Nguyen N.T.H., Phuc P.T., Huang C.-C. (2022). The Effects of Non-Pharmaceutical Interventions on COVID-19 Epidemic Growth Rate during Pre- and Post-Vaccination Period in Asian Countries. IJERPH.

[12] Mugambe R.K., Ssekamatte T., Kisaka S., Wafula S.T., Isunju J.B., Nalugya A., Oputan P., Makanga D.K., Mukiibi M., Buregyeya E. (2021). Extent of Compliance with COVID-19 Prevention and Control Guidelines among Supermarkets in Kampala Capital City and Mukono Municipality, Uganda. PLoS ONE.

[13] Petherick A., Goldszmidt R., Andrade E.B., Furst R., Hale T., Pott A., Wood A. (2021). A Worldwide Assessment of Changes in Adherence to COVID-19 Protective Behaviours and Hypothesized Pandemic Fatigue. Nat. Hum. Behav..

[14] Kim J., Hong K., Yum S., Gómez Gómez R.E., Jang J., Park S.H., Choe Y.J., Ryu S., Park D.W., Lee Y.S. (2021). Factors Associated with the Difference between the Incidence and Case-Fatality Ratio of Coronavirus Disease 2019 by Country. Sci. Rep..

[15] Liang L.-L., Kao C.-T., Ho H.J., Wu C.-Y. (2021). COVID-19 Case Doubling Time Associated with Non-Pharmaceutical Interventions and Vaccination: A Global Experience. J. Glob. Health.

[16] Paital B., Agrawal P.K. (2021). Air Pollution by NO_2_ and PM_2.5_ Explains COVID-19 Infection Severity by Overexpression of Angiotensin-Converting Enzyme 2 in Respiratory Cells: A Review. Environ. Chem. Lett..

[17] Abdullah F., Myers J., Basu D., Tintinger G., Ueckermann V., Mathebula M., Ramlall R., Spoor S., de Villiers T., der Walt Z.V. (2022). Decreased Severity of Disease during the First Global Omicron Variant COVID-19 Outbreak in a Large Hospital in Tshwane, South Africa. Int. J. Infect. Dis..

[18] Elbe S., Buckland-Merrett G. (2017). Data, Disease and Diplomacy: GISAID’s Innovative Contribution to Global Health: Data, Disease and Diplomacy. Glob. Chall..

[19] Ritchie H., Mathieu E., Rodés-Guirao L., Appel C., Giattino C., Ortiz-Ospina E., Hasell J., Macdonald B., Beltekian D., Roser M. Coronavirus Pandemic (COVID-19). https://ourworldindata.org/coronavirus.

[20] Hale T., Angrist N., Goldszmidt R., Kira B., Petherick A., Phillips T., Webster S., Cameron-Blake E., Hallas L., Majumdar S. (2021). A Global Panel Database of Pandemic Policies (Oxford COVID-19 Government Response Tracker). Nat. Hum. Behav..

[21] Mathieu E., Ritchie H., Ortiz-Ospina E., Roser M., Hasell J., Appel C., Giattino C., Rodés-Guirao L. (2021). A Global Database of COVID-19 Vaccinations. Nat. Hum. Behav..

[22] World Development Indicators World Development Indicators—World Bank Collection. http://data.worldbank.org/data-catalog/world-development-indicators.

[23] Institute for Health Metrics and Evalution (2022). GBD Results Tool.

[24] Bell J.A., Jennifer B. Nuzzo GHS Index Report and Data. https://www.ghsindex.org/report-model/.

[25] World Health Organization (2016). Global Strategy on Human Resources for Health: Workforce 2030.

[26] UK Health Security Agency COVID-19 Variants Identified in the UK. https://www.gov.uk/government/news/covid-19-variants-identified-in-the-uk.

[27] Li H., Wang L., Zhang M., Lu Y., Wang W. (2022). Effects of Vaccination and Non-Pharmaceutical Interventions and Their Lag Times on the COVID-19 Pandemic: Comparison of Eight Countries. PLoS Negl. Trop. Dis..

[28] Bates D., Mächler M., Bolker B., Walker S. (2015). Fitting Linear Mixed-Effects Models Using Lme4. J. Stat. Soft..

[29] Christensen P.A., Olsen R.J., Long S.W., Snehal R., Davis J.J., Ojeda Saavedra M., Reppond K., Shyer M.N., Cambric J., Gadd R. (2022). Signals of Significantly Increased Vaccine Breakthrough, Decreased Hospitalization Rates, and Less Severe Disease in Patients with Coronavirus Disease 2019 Caused by the Omicron Variant of Severe Acute Respiratory Syndrome Coronavirus 2 in Houston, Texas. Am. J. Pathol..

[30] Burki T.K. (2021). Challenges in the Rollout of COVID-19 Vaccines Worldwide. Lancet Respir. Med..

[31] Bollyky T.J., Hulland E.N., Barber R.M., Collins J.K., Kiernan S., Moses M., Pigott D.M., Reiner Jr R.C., Sorensen R.J.D., Abbafati C. (2022). Pandemic Preparedness and COVID-19: An Exploratory Analysis of Infection and Fatality Rates, and Contextual Factors Associated with Preparedness in 177 Countries, from Jan 1, 2020, to Sept 30, 2021. Lancet.

[32] Mendonca D., Fiedrich F. (2006). Training for Improvisation in Emergency Management: Opportunities and Limits for Information Technology. Int. J. Emerg. Manag..

[33] Geng J., Yu X., Bao H., Feng Z., Yuan X., Zhang J., Chen X., Chen Y., Li C., Yu H. (2021). Chronic Diseases as a Predictor for Severity and Mortality of COVID-19: A Systematic Review with Cumulative Meta-Analysis. Front. Med..

[34] Campi I., Gennari L., Merlotti D., Mingiano C., Frosali A., Giovanelli L., Torlasco C., Pengo M.F., Heilbron F., Soranna D. (2021). Vitamin D and COVID-19 Severity and Related Mortality: A Prospective Study in Italy. BMC Infect. Dis..

[35] Mariani J., Giménez V.M.M., Bergam I., Tajer C., Antonietti L., Inserra F., Ferder L., Manucha W. (2021). Association between Vitamin D Deficiency and COVID-19 Incidence, Complications, and Mortality in 46 Countries: An Ecological Study. Health Secur..

[36] De Andrade M.I.S., de Macêdo P.F.C., de Oliveira T.L.P.S., da Silva Lima N.M., da Costa Ribeiro I., Santos T.M. (2020). Vitamin A and D Deficiencies in the Prognosis of Respiratory Tract Infections: A Systematic Review with Perspectives for COVID-19 and a Critical Analysis on Supplementation.

[37] Tepasse P.-R., Vollenberg R., Fobker M., Kabar I., Schmidt H., Meier J.A., Nowacki T., Hüsing-Kabar A. (2021). Vitamin A Plasma Levels in COVID-19 Patients: A Prospective Multicenter Study and Hypothesis. Nutrients.

[38] Fiasca F., Minelli M., Maio D., Minelli M., Vergallo I., Necozione S., Mattei A. (2020). Associations between COVID-19 Incidence Rates and the Exposure to PM_2.5_ and NO_2_: A Nationwide Observational Study in Italy. Int. J. Environ. Res. Public Health.

