# Supplementary material for: Comparison of COVID-19 Resilience Index and Its Associated Factors across 29 Countries during the Delta and Omicron Variant Periods
Source: Vaccines (Basel). 2022 Jun 13;10(6):940. doi: 10.3390/vaccines10060940 (PMC9228202; doi:10.3390/vaccines10060940)
Supplement: Supplementary file 1 [file vaccines-10-00940-s001.zip › vaccines-1715658-supplementary.pdf]

Supplemental material

Comparison of COVID-19 Resilience Index and Its Associated Factors Across 29 Countries During the Delta and Omicron Variants Periods

Table of Contents

1. Model formulas for longitudinal analysis 4

2. Model selection procedure and testing assumptions of model 4

  2.1. Delta variant dominant period 5

  2.2. Omicron variant dominant period 8

3. Variables and data sources 12

4. Study period 14

5. Vaccination, stringency index and resilience index 15

6. Unstandardized average number of Hospitalization, ICU and mortality rate throughout the Delta and Omicron period 21

List of Tables

Table S1. Univariate model results and sequence in which covariate were added in the forward selection multivariable linear mixed model during Delta variant dominant period 5

Table S2. The final model of multivariable linear mixed effect model in Delta variant dominant period 6

Table S3. The variance inflation factor (VIF) for covariates in the mLME during Delta variant dominant period 8

Table S4. Univariate model results and sequence in which covariates were added in the forward selection multivariable linear mixed effect model (mLME) during Omicron variant dominant period 8

Table S5. The final model of multivariable linear mixed effect model in Omicron variant dominant period 10

Table S6. The variance inflation factor (VIF) for covariates in the mLME during Omicron variant dominant period 12

Table S7. List of variables and their data sources 12

Table S8. The study period of countries during Delta variant and Omicron variant period 14

Table S9. The variation of stringency index in 29 countries during the first eight weeks of the Delta period 15

Table S10. The variation of stringency index in 29 countries during the first eight weeks of the Omicron period 16

Table S11. The percentage of population fully vaccinated in 29 countries during the first eight weeks of the Delta period 17

Table S12. The percentage of population fully vaccinated in 29 countries during the first eight weeks of the Omicron period 18

Table S13. The variation of standardized resilience index in 29 countries during the first eight weeks of the Delta period 19

Table S14. The variation of standardized resilience index in 29 countries during the first eight weeks of the Omicron period 20

Table S15. The unstandardized average number of daily hospital occupancy, daily ICU occupancy and mortality rate per 100000 inhabitants 21

List of Figures

Figure S1. The residuals across all countries and over the time for the mLME during Delta variant dominant period 7

Figure S2. The residuals in the intercepts and slopes across countries and in the between-country intercept and slope residuals for mLME during Delta variant dominant period. .. 8

Figure S3. The residuals across all countries and over the time for the mLME during Omicron variant dominant period. 11

Figure S4. The residuals in the intercepts and slopes across countries and in the between-country intercept and slope residuals for mLME during Omicron variant dominant period. 12

1. Model formulas for longitudinal analysis

$$\boldsymbol{Y_{i,t} = \beta_0 + (\alpha + \alpha_i)T_{i,t} + \sum_{j=1}^{n} \beta_j X_{ij,t} + (\theta_0 P_{i,t}) + (\delta_0 V_{i,t}) + b_i + \varepsilon_{i,t}}$$

Our interest outcome $Y_{i,t}$ is the log transformation of resilience index (r index) in the country i on the week t.

The corresponding independent variables that were classified into three equal group and significantly associated with outcome variable in univariate analysis are denoted by $X_{ij,t}$(j=1,…,n with n – number of selected independent variables by the forward selection approach) their fixed effects are captured by β_j.

$P_{i,t}$ is the categorical variable (tertiles) for 14-day lag stringency index of country i on week t. Their fixed effect is captured by θ_0.

$V_{i,t}$ is the categorical variable (tertiles) for 40-day lag of percentage of population fully vaccinated in country i on week t. The effects of vaccination coverage is captured via the fixed effect δ_0.

The time of NPI implementation (measured by stringency index) or vaccination is denoted by $T_{i,t}$ and its effect is captured via fixed effect α.

The random intercept model was used because it enabled each country to have its own linear regression function with different intercept (corresponding to fixed intercept β_0). Country-specific random slopes for week $T_{i,t}$ might be important if the resilience index has varied by different weeks. Therefore, we extended the mixed models that allows the country-specific random slopes for week effect $T_{i,t}$ with a random effect α_i. Our model parameters are estimated via maximum likelihood (ML) approach.

The country-specific impact was captured via the random effect b_i ~ N (0,D). $\varepsilon_{i,t}$~ N (0, σ_e^2) was the country and week specific residual error.

D is the variance-covariance matrix for the intercept and slope pertaining to the outcome Y.

2. Model selection procedure and testing assumptions of model

According to forward selection approach, the steps to select the final model include:

Step 1: The univariate analysis linear mixed effect models were conducted with time and each covariate (stringency index, percentage of population fully vaccinated, independent variables) as predictors

Step 2: We selected all the covariates which had significant value (p value <0.05) in the univariate analyses.

Step 3: We ranked the selected covariate in the decreasing order according to the goodness of fit of the univariate models as defined by Bayesian Information Criterion (BIC).

Step 4: We fitted a series of multivariable forward selection linear mixed models with time and inputted each covariate sequentially based on its rank. If an individual variable was an insignificant predictor, it was dropped from the forward selection models.

Step 5: After achieving the final model for linear mixed effect model, we fitted the generalized linear mixed model with the similar covariates.

2.1. Delta variant dominant period

2.1.1. Model selection of the multivariable linear mixed effect model (mLME) during Delta variant dominant period

We identified the significant covariates using the univariate analysis. Table S1 show the selected covariates were ranked in decreasing order of good of fitness based on their BIC values.

Table S1. Univariate model results and sequence in which covariate were added in the forward selection multivariable linear mixed model during Delta variant dominant period

**Rank**	**Covariates**	**Coefficient**	**p value**	**BIC**
1	Intercept	-3.655	0.000	191.696
	Percentage of population fully vaccinated (T2)	-0.294	0.000	
	Percentage of population fully vaccinated (T3)	-0.221	0.042	
	Time	0.163	0.000	
2	Intercept	-3.87	0.000	199.017
	Stringency index (T2)	0.072	0.414	
	Stringency index (T3)	0.272	0.006	
	Time	0.148	0.000	
3	Intercept	-3.166	0.000	201.929
	Regulatory Quality (T2)	-0.504	0.13	
	Regulatory Quality (T3)	-1.243	0.000	
	Time	0.147	0.000	
4	Intercept	-3.335	0.000	207.985
	Rule of Law (T2)	-0.323	0.376	
	Rule of Law (T3)	-0.916	0.014	
	Time	0.147	0.000	
5	Intercept	-3.231	0.000	208.449

	No. Nurses per 1000 inhabitants (T2)	-0.831	0.029	
	No. Nurses per 1000 inhabitants (T3)	-0.76	0.039	
	Time	0.147	0.000	
6	Intercept	-3.491	0.000	208.780
	Government Effectiveness (T2)	0.004	0.991	
	Government Effectiveness (T3)	-0.76	0.041	
	Time	0.147	0.000	
7	Intercept	-4.175	0.000	209.133
	Population (T2)	0.41	0.272	
	Population (T3)	0.859	0.023	
	Time	0.147	0.000	

Table S2 presents the multivariable linear mixed effect model (mLME) which includes the significant covariates selected from the forward selection during Delta variant dominant period.

Table S2. The final model of multivariable linear mixed effect model in Delta variant dominant period

**Covariates**	**Coefficient**	**p values**	**Lower bound**	**Upper bound**
Intercept	-3.620	0.000	-4.312	-2.928
Medium level -% population fully vaccinated (T2)	-0.300	0.000	-0.433	-0.167
High level -% population fully vaccinated (T3)	-0.233	0.033	-0.447	-0.019
Medium level - Rule of Law (T2)	-0.279	0.447	-1.023	0.465
High level - Rule of Law (T3)	-0.892	0.018	-1.620	-0.164
Medium level - Population (T2)	0.350	0.341	-0.394	1.094
Medium level - Population (T3)	0.730	0.049	0.003	1.456
Time	0.164	0.000	0.122	0.206
BIC	218.844			
R2 Conditional	0.957			
R2 Marginal	0.344			

2.1.2. Testing the assumption of the mLME during Delta variant dominant period

- Normality in the distribution of the residuals

To examine the normality of residual distribution, we plotted the residuals across all countries and over time in figure S1; and the residuals in the intercepts and slopes across countries and the between-country intercept and slope residuals during the Delta variant dominant period in figure S2.

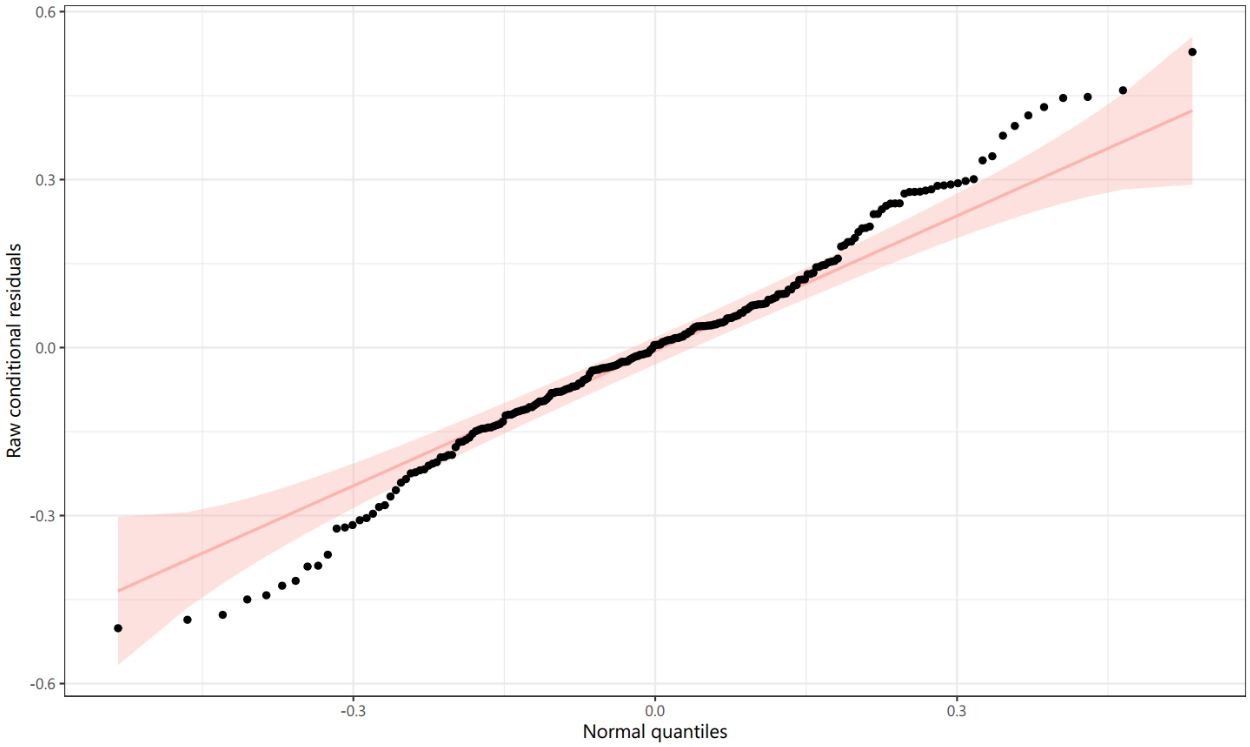

Figure S1. The residuals across all countries and over the time for the mLME during Delta variant dominant period

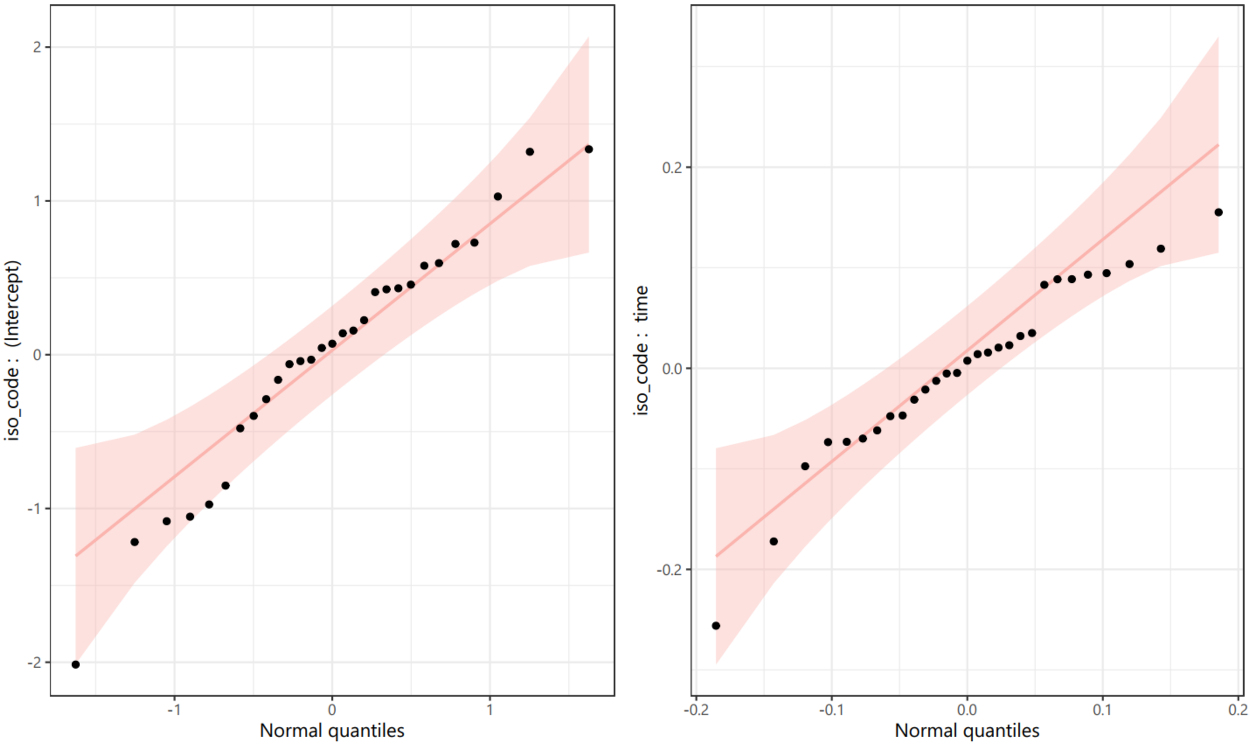

Figure S2. The residuals in the intercepts and slopes across countries and in the between-country intercept and slope residuals for mLME during Delta variant dominant period.

Based on results in the figure S1 and figure S2, we can conclude that the distribution of these residuals can be considered approximately normal.

- Low collinearity between predictors

Table S3. The variance inflation factor (VIF) for covariates in the mLME during Delta variant dominant period

**Covariates**	**VIF**
Percentage of population fully vaccinated (Tertiles)	1.247
Rule of Law (Tertiles)	1.017
Population (Tertiles)	1.013
Time	1.240

The variance inflation factor (VIF) for independent variables in the mLME are presented in Table S3. VIF values are about 1 which means lack of strong collinearity

2.2. Omicron variant dominant period

2.2.1. Model selection of the multivariable linear mixed effect model (mLME) during Omicron variant dominant period

Table S4 shows the significant covariates (p<0.05) was identified using the univariate analysis of mLME. The variables were ranked in decreasing order of good of fitness based on their BIC values.

Table S4. Univariate model results and sequence in which covariates were added in the forward selection multivariable linear mixed effect model (mLME) during Omicron variant dominant period

**Rank**	**Covariates**	**Coefficient**	**P values**	**BIC**
1	Intercept	-1.35	0.000	146.445
	Government Effectiveness (T2)	-0.373	0.094	
	Government Effectiveness (T3)	-0.972	0.000	
	Time	0.069	0.002	
2	Intercept	-1.372	0.000	146.808
	% population fully vaccinated (T2)	-0.649	0.000	
	% population fully vaccinated (T3)	-0.699	0.000	
	Time	0.074	0.003	

3	Intercept	-1.497	0.000	149.207
	Rule of Law (T2)	-0.067	0.769	
	Rule of Law (T3)	-0.822	0.001	
	Time	0.069	0.002	
4	Intercept	-1.989	0.000	151.369
	Number of hospital beds per 1000 inhabitants (T2)	-0.136	0.567	
	Number of hospital beds per 1000 inhabitants (T3)	0.667	0.007	
	Time	0.069	0.002	
5	Intercept	-1.423	0.000	152.488
	Regulatory Quality (T2)	-0.338	0.168	
	Regulatory Quality (T3)	-0.792	0.002	
	Time	0.069	0.002	
6	Intercept	-2.267	0.000	152.820
	Burden of cancer (T2)	0.662	0.01	
	Burden of cancer (T3)	0.757	0.003	
	Time	0.069	0.002	
7	Intercept	-1.466	0.000	152.864
	% population living in Urban area (T2)	-0.213	0.382	
	% population living in Urban area (T3)	-0.779	0.002	
	Time	0.069	0.002	
8	Intercept	-1.42	0.000	153.764
	GDP per capita (T2)	-0.399	0.113	
	GDP per capita (T3)	-0.746	0.004	
	Time	0.069	0.002	
9	Intercept	-2.275	0.000	154.586
	Burden of tobacco smoking (T2)	0.652	0.013	
	Burden of tobacco smoking (T3)	0.788	0.003	
	Time	0.069	0.002	
10	Intercept	-1.518	0.000	156.494
	Burden of chronic kidney diseases (T2)	-0.643	0.018	
	Burden of chronic kidney diseases (T3)	-0.242	0.34	
	Time	0.069	0.002	
11	Intercept	-2.157	0.000	156.612
	Burden of low bone density (T2)	0.421	0.111	
	Burden of low bone density (T3)	0.654	0.014	
	Time	0.069	0.002	
12	Intercept	-1.447	0.000	156.655
	Life expectancy (T2)	-0.574	0.033	
	Life expectancy (T3)	-0.509	0.051	
	Time	0.069	0.002	
13	Intercept	-1.98	0.000	156.711
	Burden of non-communicable diseases (T2)	-0.015	0.954	
	Burden of non-communicable diseases (T3)	0.534	0.041	

	Time	0.069	0.002	
14	Intercept	-1.614	0.000	156.861
	Population density (T2)	-0.006	0.981	
	Population density (T3)	-0.537	0.041	
	Time	0.069	0.002	
15	Intercept	-2.086	0.000	157.152
	Burden of cardiovascular diseases (T2)	0.246	0.349	
	Burden of cardiovascular diseases (T3)	0.605	0.023	
	Time	0.069	0.002	
16	Intercept	-2.118	0.000	157.241
	Burden of zinc deficiency (T2)	0.363	0.171	
	Burden of zinc deficiency (T3)	0.592	0.026	
	Time	0.069	0.002	

Table S5 presents the results of the multivariable linear mixed effect model (mLME) which includes the significant covariates selected from the forward selection during Omicron variant dominant period.

Table S5. The final model of multivariable linear mixed effect model in Omicron variant dominant period

**Covariates**	**Coefficient**	**p value**	**Lower bound**	**Upper bound**
Intercept	-1.695	0.000	-2.182	-1.207
Medium level -% population fully vaccinated (T2)	-0.306	0.041	-0.600	-0.012
High level -% population fully vaccinated (T3)	-0.358	0.040	-0.700	-0.017
Medium level - Government effectiveness (T2)	-0.338	0.037	-0.654	-0.022
High level - Government effectiveness (T3)	-0.780	0.000	-1.118	-0.443
Medium burden -Low bone density (T2)	0.314	0.082	-0.042	0.670
High burden -Low bone density (T3)	0.531	0.003	0.191	0.871
Medium level - Death rate caused by Cancer (T2)	0.430	0.016	0.088	0.773
High level - Death rate caused by Cancer (T3)	0.183	0.311	-0.179	0.544
Time	0.072	0.002	0.028	0.116
BIC	154.821			
R2 Conditional	0.927			
R2 Marginal	0.623			

2.2.2. Testing the assumption of the mLME during Omicron variant dominant period

- Normality in the distribution of the residuals

The figure S3 presents the residuals across all countries and over the time.

The residuals in the intercepts and slopes across countries and in the between-country intercept and slope residuals were indicated in the Figure S4.

According to results of Figure S3 and Figure S4, the distribution of these residuals can be considered approximately normal.

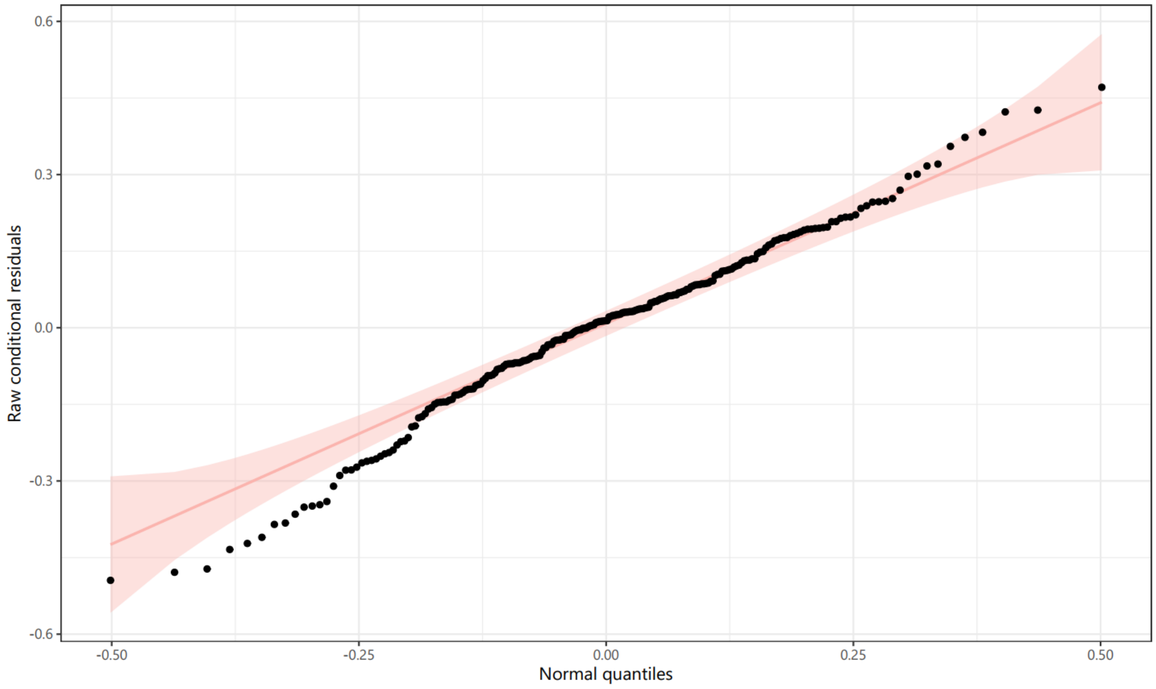

Figure S3. The residuals across all countries and over the time for the mLME during Omicron variant dominant period.

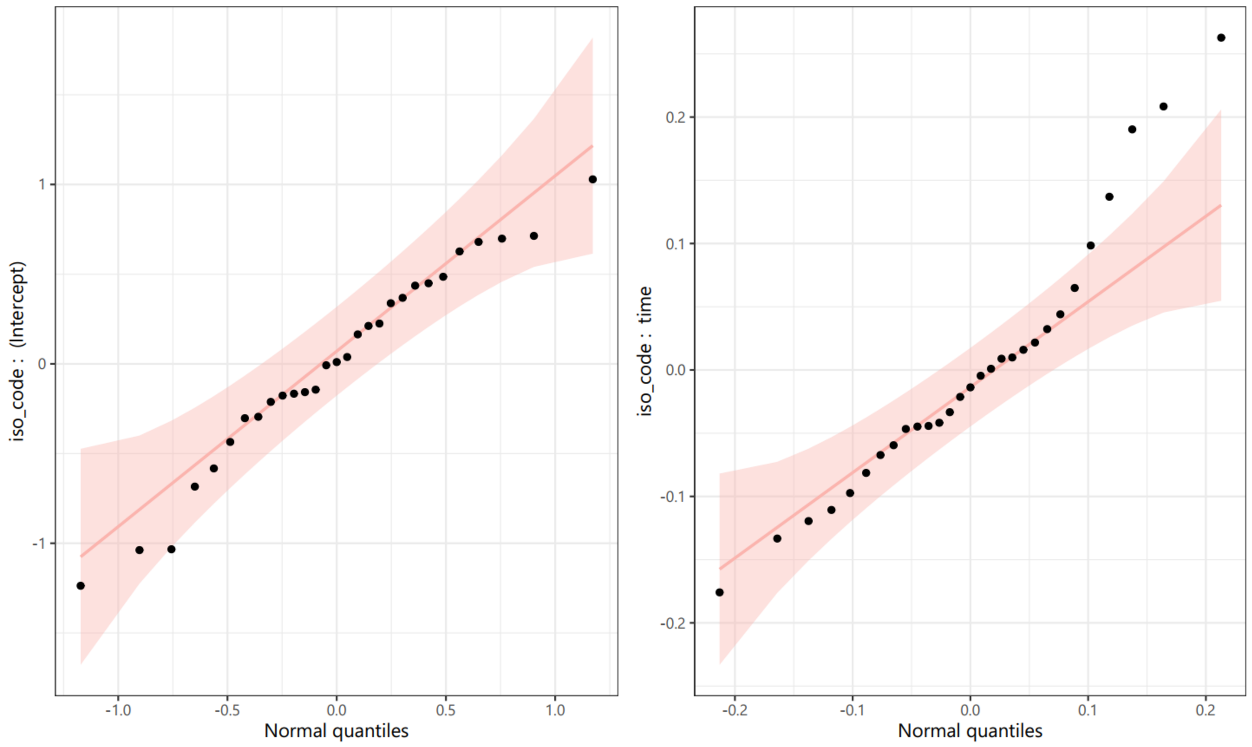

Figure S4. The residuals in the intercepts and slopes across countries and in the between-country intercept and slope residuals for mLME during Omicron variant dominant period.

- Low collinearity between predictors

Table S6. The variance inflation factor (VIF) for covariates in the mLME during Omicron variant dominant period

**Covariates**	**VIF**
Percentage of population fully vaccinated (Tertiles)	1.863
Government effectiveness (Tertiles)	1.438
Burden of Low bone density (Tertiles)	1.528
Burden of cancer (Tertiles)	1.747
Time	1.009

Table S6 indicates the variance inflation factor (VIF) for covariates in the mLME. VIF values were below 10, which means a lack of strong collinearity.

3. Variables and data sources

Table S7. List of variables and their data sources

	Variables	Range value	Values recorded	Data source
1	Rule of Law	1 - 5	No applicable	Worldwide Governance Indicators<br>http://info.worldbank.org/governance/wgi
2	Regulatory Quality	1 -5	No applicable	
3	Government Effectiveness	-2.5 - 2.5	No applicable	
4	Population	No applicable	No applicable	World Bank Open Data<br>Link: https://data.worldbank.org/
5	Population density	No applicable	No applicable	
6	Life expectancy	No applicable	No applicable	
7	GDP per capita (USD per capita)	No applicable	No applicable	
8	% population aged over 65	No applicable	No applicable	
9	% population living in Urban area	No applicable	No applicable	
10	UHC index	0-100	No applicable	Global Health Observatory indicator views<br>https://apps.who.int/gho/data/node.imr 2019
11	No. Physician per 1000	No applicable	No applicable	World Bank Open Data<br>https://data.worldbank.org
12	No. Nurse and Midwife per 1000	No applicable	No applicable	
13	% GDP for health expenditure	No applicable	No applicable	
14	PM2.5	No applicable	No applicable	
15	Tobacco	No applicable	No applicable	
16	Global heath security index (GHS index)	No applicable	No applicable	World Health Organization<br>https://www.ghsindex.org/about/
17	Non-communicable diseases	No applicable	No applicable	Global Burden of disease<br>https://www.healthdata.org/gbd/2019<br>(We retrieved the death rate per 100000 inhabitants and years live with disability (YLD) per 100000 inhabitants)
18	Diabetes	No applicable	No applicable	
19	Chronic respiratory disease	No applicable	No applicable	
20	Cancer	No applicable	No applicable	
21	Chronic kidney diseases	No applicable	No applicable	
22	Cardiovascular diseases	No applicable	No applicable	
23	Zinc deficiency	No applicable	No applicable	
24	Vitamin A deficiency	No applicable	No applicable	
25	Low bone density	No applicable	No applicable	
26	Stringency index	0 - 100	No applicable	Oxford database: The Oxford COVID-19 Government Response Tracker (OxCGRT)<br>Link:<br>https://www.bsg.ox.ac.uk/research/research-projects/covid-19-government-response-tracker

27	% population vaccinated at least one dose	No applicable	No applicable	Oxford database: A global database of COVID-19 vaccinations<br><br>https://ourworldindata.org/covid-vaccinations 2021
28	% population fully vaccinated	No applicable	No applicable	
29	% population vaccinated the booster dose	No applicable	No applicable	

4. Study period

Table S8. The study period of countries during Delta variant and Omicron variant period

ISO Code	Country	The first date of study period (Delta)	The last date of study period (Delta)	The first date of study period (Omicron)	The last date of study period (Omicron)
AUS	Australia	06/20/2021	08/14/2021	12/26/2021	02/19/2022
BEL	Belgium	06/27/2021	08/21/2021	01/02/2022	02/26/2022
BGR	Bulgaria	07/04/2021	08/28/2021	01/16/2022	03/12/2022
CAN	Canada	07/11/2021	09/04/2021	12/19/2021	02/12/2022
CZE	Czechia	06/27/2021	08/21/2021	01/02/2022	02/26/2022
CHE	Switzerland	07/04/2021	08/28/2021	12/26/2021	02/19/2022
DNK	Denmark	07/04/2021	08/28/2021	12/26/2021	02/19/2022
ESP	Spain	07/04/2021	08/28/2021	12/26/2021	02/19/2022
EST	Estonia	07/04/2021	08/28/2021	01/09/2022	03/05/2022
FIN	Finland	06/20/2021	08/14/2021	01/02/2022	02/26/2022
FRA	France	07/04/2021	08/28/2021	01/02/2022	02/26/2022
GBR	United Kingdom	05/23/2021	07/17/2021	12/19/2021	02/12/2022
IRL	Ireland	06/27/2021	08/21/2021	12/19/2021	02/12/2022
ISR	Israel	06/06/2021	07/31/2021	12/26/2021	02/19/2022
ITA	Italy	07/04/2021	08/28/2021	01/02/2022	02/26/2022
JPN	Japan	07/25/2021	09/18/2021	01/02/2022	02/26/2022
LUX	Luxembourg	06/20/2021	08/14/2021	01/02/2022	02/26/2022
LVA	Latvia	06/27/2021	08/21/2021	01/16/2022	03/12/2022
MLT	Malta	07/04/2021	08/28/2021	01/02/2022	02/26/2022
MYS	Malaysia	06/13/2021	08/07/2021	12/26/2021	02/19/2022
NLD	Netherlands	07/04/2021	08/28/2021	01/02/2022	02/26/2022
PRT	Portugal	06/13/2021	08/07/2021	12/26/2021	02/19/2022

ROU	Romania	07/11/2021	09/04/2021	01/02/2022	02/26/2022
SGP	Singapore	04/25/2021	06/19/2021	12/26/2021	02/19/2022
SRB	Serbia	07/25/2021	09/18/2021	01/02/2022	02/26/2022
SVK	Slovakia	07/11/2021	09/04/2021	01/16/2022	03/12/2022
SVN	Slovenia	07/11/2021	09/04/2021	01/02/2022	02/26/2022
SWE	Sweden	07/04/2021	08/28/2021	12/26/2021	02/19/2022
USA	United States	06/27/2021	08/21/2021	12/26/2021	02/19/2022

5. Vaccination, stringency index and resilience index

Table S9. The variation of stringency index in 29 countries during the first eight weeks of the Delta period

Country	Week 0	Week 1	Week 2	Week 3	Week 4	Week 5	Week 6	Week 7
Australia	55.4	69.1	68.1	68.1	68.1	68.1	68.1	69.6
Belgium	50.9	50.9	50.9	50.9	49.9	48.3	47.2	47.2
Bulgaria	42.6	42.6	42.6	42.6	20.4	20.4	20.4	20.4
Canada	67.3	60.7	60.7	59.1	60.7	60.7	65.1	70.7
Czechia	39.0	36.0	35.2	35.7	37.0	37.0	37.0	27.6
Denmark	47.2	47.2	47.2	46.0	38.9	38.9	38.9	38.9
Estonia	27.4	23.2	23.2	23.2	23.2	23.2	23.2	25.5
Finland	47.7	46.1	44.0	44.0	44.0	43.2	38.4	38.4
France	46.2	44.0	44.0	44.0	44.0	56.7	66.7	66.7
Ireland	50.0	44.4	44.4	44.4	44.4	44.4	44.4	44.4
Israel	29.6	24.3	25.7	29.6	29.6	29.6	33.6	35.2
Italy	47.2	63.4	75.5	70.6	59.9	62.3	63.9	63.9
Japan	50.5	50.5	50.5	50.7	52.3	52.3	52.3	51.0
Latvia	41.7	41.7	41.7	41.7	41.7	41.7	41.7	41.7
Luxembourg	41.7	41.7	41.7	41.7	39.0	38.0	38.0	38.0
Malaysia	74.7	78.7	79.5	80.6	80.6	83.9	85.2	83.3
Malta	44.3	45.6	47.2	47.2	46.2	43.5	43.5	43.5
Netherlands	32.9	36.1	40.1	41.7	41.7	41.7	41.7	41.7
Portugal	64.4	65.6	67.7	66.4	63.9	63.9	63.9	58.3
Romania	36.4	38.4	38.4	38.4	40.8	55.1	55.1	55.1

Serbia	36.1	36.1	36.1	36.1	36.1	36.1	36.1	36.1
Singapore	50.9	52.3	52.8	56.0	58.3	58.3	58.3	58.3
Slovakia	44.0	44.0	44.0	44.0	44.0	36.0	34.7	34.7
Slovenia	28.6	25.9	25.9	27.0	29.6	33.6	35.2	37.8
Spain	48.6	48.9	48.5	47.7	47.7	47.7	47.7	47.7
Sweden	51.9	45.5	37.0	37.0	37.0	37.0	37.0	37.0
Switzerland	44.4	44.4	42.3	40.7	40.7	40.7	40.7	40.7
United Kingdom	62.5	62.5	51.4	51.4	51.4	51.4	51.4	51.4
United States	61.6	56.4	49.5	49.5	49.5	49.5	54.2	56.0

Table S10. The variation of stringency index in 29 countries during the first eight weeks of the Omicron period

**Country**	**Week 0**	**Week 1**	**Week 2**	**Week 3**	**Week 4**	**Week 5**	**Week 6**	**Week 7**
Australia	40.3	41.9	44.2	61.3	64.2	55.6	55.6	53.2
Belgium	44.4	44.4	44.4	44.4	50.0	50.0	50.0	47.4
Bulgaria	50.0	50.0	46.0	44.4	44.4	44.4	44.4	44.4
Canada	67.4	71.9	77.7	78.2	76.7	78.2	77.7	77.2
Czechia	38.0	38.0	47.2	47.2	42.5	41.7	41.7	41.7
Denmark	38.4	35.2	37.8	38.9	38.9	23.0	16.7	16.7
Estonia	42.6	43.5	43.5	49.1	49.5	46.3	46.3	46.3
Finland	34.7	34.7	34.7	40.7	42.1	41.7	41.7	41.7
France	72.2	69.4	69.4	69.4	69.4	69.4	69.4	69.4
Ireland	50.4	52.8	52.8	52.8	48.5	23.2	23.2	23.2
Israel	56.5	56.5	56.5	56.5	56.5	56.5	49.3	48.2
Italy	76.9	76.9	76.9	76.9	76.9	76.9	76.9	76.9
Japan	47.2	47.2	47.2	47.2	47.2	47.2	47.2	47.2
Latvia	35.2	37.0	48.2	48.2	48.2	48.2	44.4	37.0
Luxembourg	46.3	46.3	46.3	52.9	55.6	50.3	46.3	46.3
Malaysia	50.5	50.5	62.8	64.8	64.8	54.2	54.2	54.2
Malta	43.5	43.5	43.5	46.2	52.8	52.8	52.8	52.8
Netherlands	69.4	65.2	58.3	58.3	58.3	58.3	50.4	47.2

Portugal	45.0	42.6	35.7	31.5	31.5	31.5	31.5	29.9
Romania	52.8	52.8	52.8	52.8	48.8	47.2	47.2	47.2
Serbia	28.7	28.7	28.7	34.0	36.1	36.1	36.1	36.1
Singapore	44.4	44.4	44.4	55.7	60.2	60.2	60.2	60.2
Slovakia	54.4	50.5	49.5	46.3	46.3	46.3	46.3	46.3
Slovenia	51.3	50.0	61.1	63.0	63.0	63.0	62.4	59.3
Spain	54.2	43.5	43.5	43.5	46.7	49.1	49.1	49.1
Sweden	49.1	49.1	51.2	52.4	50.0	50.0	32.5	19.4
Switzerland	50.9	56.5	56.5	56.5	56.5	56.5	56.5	42.2
United Kingdom	48.6	48.6	48.6	48.6	48.1	44.8	42.1	41.3
United States	50.1	50.5	50.5	53.6	56.0	58.0	58.8	58.8

Table S11. The percentage of population fully vaccinated in 29 countries during the first eight weeks of the Delta period

**Country**	**Week 0**	**Week 1**	**Week 2**	**Week 3**	**Week 4**	**Week 5**	**Week 6**	**Week 7**
Australia	5.6	7.2	8.9	10.9	11.0	15.2	17.9	20.8
Belgium	36.5	42.3	48.1	54.0	59.6	61.1	66.4	68.2
Bulgaria	12.3	12.4	12.4	14.5	15.0	15.5	16.0	16.7
Canada	49.1	54.6	58.8	61.4	63.4	65.2	66.6	67.6
Czechia	32.8	35.8	39.6	43.0	45.5	47.6	49.8	51.7
Denmark	38.5	43.6	49.0	53.4	57.8	63.6	67.6	70.0
Estonia	37.0	39.1	41.2	42.6	44.0	45.4	46.5	47.6
Finland	15.5	17.8	21.0	24.7	28.0	31.7	35.5	39.1
France	33.5	41.4	45.2	48.0	50.2	52.3	55.6	58.8
Ireland	40.0	45.8	49.8	53.8	57.0	59.8	63.0	65.9
Israel	55.6	55.7	55.8	56.0	56.0	56.5	57.3	58.2
Italy	38.0	43.4	48.3	52.5	55.5	56.5	58.5	60.2
Japan	30.0	33.0	36.8	40.1	43.9	47.3	50.0	53.3
Latvia	30.9	33.1	34.3	35.5	36.6	37.6	38.5	39.5
Luxembourg	6.0	6.0	6.0	6.0	6.0	6.0	6.0	6.0
Malaysia	4.9	6.3	7.9	10.7	13.8	16.9	21.1	26.5
Malta	67.8	69.2	71.6	74.1	76.6	78.4	79.3	79.8

Netherlands	41.2	46.3	50.2	54.1	58.4	62.2	63.5	64.7
Portugal	28.7	32.5	34.6	40.3	48.3	53.4	59.0	64.3
Romania	24.7	25.1	25.4	25.8	25.8	26.4	26.7	27.0
Serbia	39.3	39.6	40.1	40.3	40.7	41.0	41.2	41.6
Singapore	20.8	22.6	25.2	28.3	31.0	33.4	35.5	37.3
Slovakia	35.3	36.5	37.7	39.0	40.0	40.8	41.5	42.0
Slovenia	38.0	39.2	40.3	41.4	42.5	43.6	44.5	45.4
Spain	44.9	50.0	54.3	57.6	60.5	63.2	66.5	69.4
Sweden	33.9	35.9	37.5	39.5	42.1	45.0	48.3	52.4
Switzerland	42.8	46.2	48.8	50.3	51.3	52.2	53.1	53.9
United Kingdom	37.1	38.2	43.7	46.0	47.6	49.3	51.0	51.1
United States	49.2	49.7	50.2	50.7	51.2	51.7	52.2	53.0

Table S12. The percentage of population fully vaccinated in 29 countries during the first eight weeks of the Omicron period

**Country**	**Week 0**	**Week 1**	**Week 2**	**Week 3**	**Week 4**	**Week 5**	**Week 6**	**Week 7**
Australia	76.6	77.1	77.6	78.0	78.0	78.7	79.0	79.1
Belgium	76.0	76.1	76.2	76.2	76.7	77.3	77.6	78.1
Bulgaria	28.7	28.9	29.2	29.4	29.4	29.6	29.7	29.8
Canada	77.1	77.3	77.5	77.9	78.3	78.9	79.7	79.9
Czechia	62.5	62.7	63.0	63.2	63.5	63.6	63.7	63.7
Denmark	78.1	79.5	80.3	80.8	81.1	81.2	81.3	81.4
Estonia	62.4	62.6	62.8	63.0	63.1	63.2	63.3	63.4
Finland	73.6	73.6	73.6	74.4	75.3	75.8	76.0	76.1
France	74.4	75.2	75.8	76.1	76.8	76.9	77.4	77.5
Ireland	77.3	77.6	77.7	77.8	77.9	78.1	78.7	79.1
Israel	63.9	64.3	64.8	65.3	65.3	65.7	65.8	65.9
Italy	74.6	75.1	75.6	75.7	76.9	77.7	78.0	78.6
Japan	78.3	78.9	79.0	79.1	79.2	79.3	79.3	79.5
Latvia	68.7	68.8	69.2	69.3	69.5	69.6	69.6	69.6
Luxembourg	6.0	6.0	6.0	6.0	6.0	6.0	6.0	71.9
Malaysia	78.2	78.3	78.4	78.4	78.5	78.5	78.5	78.5

Malta	84.7	85.3	86.0	86.8	87.6	88.9	89.1	89.7
Netherlands	71.5	71.6	71.7	71.8	71.8	71.8	71.9	71.9
Portugal	89.5	89.8	90.1	90.3	90.5	90.6	91.3	91.4
Romania	41.2	41.4	41.5	41.7	41.8	41.9	42.0	42.1
Serbia	46.9	47.0	47.1	47.2	47.3	47.4	47.4	47.5
Singapore	85.1	85.4	85.6	86.0	86.7	87.4	88.4	89.2
Slovakia	49.6	49.9	50.2	50.4	50.5	50.6	50.6	50.6
Slovenia	57.5	57.9	58.0	58.2	58.4	58.5	58.6	58.6
Spain	81.0	81.4	81.6	81.8	81.9	81.9	82.1	82.1
Sweden	72.2	72.4	72.6	73.2	73.6	73.9	74.1	74.3
Switzerland	66.9	67.3	67.5	67.7	67.9	68.2	68.2	68.5
United Kingdom	69.3	69.6	69.9	70.3	70.5	70.9	71.1	71.5
United States	62.8	63.2	63.5	63.8	64.1	64.4	64.7	64.9

Table S13. The variation of standardized resilience index in 29 countries during the first eight weeks of the Delta period

**Country**	**Week 0**	**Week 1**	**Week 2**	**Week 3**	**Week 4**	**Week 5**	**Week 6**	**Week 7**
Australia	0.004	0.005	0.006	0.008	0.008	0.015	0.019	0.021
Belgium	0.063	0.047	0.041	0.042	0.049	0.05	0.064	0.085
Bulgaria	0.154	0.111	0.099	0.095	0.117	0.166	0.223	0.387
Canada	0.04	0.035	0.032	0.029	0.033	0.044	0.057	0.07
Czechia	0.013	0.014	0.009	0.015	0.015	0.007	0.015	0.015
Denmark	0.012	0.014	0.013	0.017	0.015	0.023	0.023	0.031
Estonia	0.019	0.016	0.019	0.024	0.04	0.048	0.07	0.085
Finland	0.012	0.011	0.013	0.015	0.014	0.018	0.026	0.028
France	0.124	0.1	0.097	0.111	0.139	0.168	0.206	0.227
Ireland	0.025	0.023	0.029	0.032	0.039	0.045	0.057	0.071
Israel	0.019	0.013	0.013	0.012	0.012	0.02	0.025	0.037
Italy	0.03	0.026	0.026	0.033	0.043	0.048	0.072	0.08
Japan	0.067	0.087	0.113	0.139	0.159	0.173	0.174	0.156
Latvia	0.145	0.108	0.063	0.045	0.041	0.028	0.034	0.056
Luxembourg	0.016	0.011	0.018	0.037	0.048	0.046	0.062	0.072

Malaysia	0.26	0.26	0.273	0.306	0.342	0.392	0.428	0.469
Malta	0.012	0.018	0.03	0.051	0.068	0.097	0.086	0.069
Netherlands	0.028	0.025	0.036	0.054	0.064	0.067	0.067	0.072
Portugal	0.053	0.064	0.074	0.087	0.108	0.128	0.136	0.141
Romania	0.027	0.022	0.02	0.029	0.031	0.056	0.085	0.129
Serbia	0.034	0.047	0.069	0.088	0.112	0.168	0.258	0.386
Singapore	0.011	0.013	0.015	0.022	0.021	0.02	0.016	0.014
Slovakia	0.03	0.033	0.032	0.028	0.031	0.034	0.022	0.032
Slovenia	0.03	0.027	0.026	0.026	0.025	0.038	0.069	0.102
Spain	0.078	0.106	0.158	0.216	0.259	0.253	0.253	0.25
Sweden	0.024	0.017	0.018	0.015	0.017	0.019	0.025	0.032
Switzerland	0.019	0.019	0.023	0.031	0.036	0.056	0.104	0.152
United Kingdom	0.018	0.017	0.02	0.025	0.031	0.035	0.049	0.051
United States	0.076	0.082	0.103	0.136	0.191	0.266	0.344	0.427

Table S14. The variation of standardized resilience index in 29 countries during the first eight weeks of the Omicron period

Country	Week 0	Week 1	Week 2	Week 3	Week 4	Week 5	Week 6	Week 7
Australia	0.057	0.085	0.156	0.197	0.198	0.181	0.143	0.13
Belgium	0.255	0.248	0.256	0.258	0.347	0.382	0.33	0.238
Bulgaria	0.876	0.817	0.913	0.98	0.992	0.79	0.765	0.51
Canada	0.078	0.103	0.175	0.281	0.318	0.318	0.275	0.266
Czechia	0.408	0.378	0.226	0.214	0.296	0.38	0.401	0.401
Denmark	0.141	0.16	0.161	0.152	0.158	0.177	0.212	0.264
Estonia	0.193	0.210	0.200	0.235	0.323	0.358	0.372	0.44
Finland	0.12	0.119	0.119	0.113	0.147	0.142	0.137	0.134
France	0.398	0.426	0.448	0.478	0.483	0.502	0.439	0.405
Ireland	0.148	0.127	0.162	0.202	0.174	0.149	0.164	0.144
Israel	0.041	0.059	0.12	0.203	0.208	0.458	0.387	0.371
Italy	0.26	0.333	0.376	0.376	0.374	0.333	0.296	0.227
Japan	0.012	0.027	0.048	0.08	0.09	0.152	0.157	0.194
Latvia	0.426	0.441	0.5	0.521	0.547	0.641	0.694	0.675

Luxembourg	0.203	0.22	0.185	0.133	0.166	0.165	0.167	0.131
Malaysia	0.086	0.076	0.069	0.055	0.048	0.05	0.072	0.105
Malta	0.202	0.256	0.264	0.276	0.21	0.224	0.228	0.111
Netherlands	0.148	0.12	0.099	0.088	0.084	0.085	0.089	0.089
Portugal	0.131	0.143	0.181	0.224	0.241	0.267	0.265	0.253
Romania	0.174	0.193	0.252	0.332	0.483	0.606	0.612	0.547
Serbia	0.241	0.253	0.3	0.384	0.505	0.557	0.502	0.463
Singapore	0.039	0.029	0.025	0.031	0.051	0.076	0.108	0.137
Slovakia	0.51	0.376	0.365	0.415	0.459	0.491	0.501	0.46
Slovenia	0.477	0.454	0.464	0.509	0.563	0.581	0.54	0.471
Spain	0.241	0.292	0.357	0.381	0.393	0.385	0.376	0.366
Sweden	0.078	0.098	0.132	0.154	0.172	0.23	0.227	0.223
Switzerland	0.272	0.251	0.245	0.238	0.222	0.212	0.213	0.186
United Kingdom	0.129	0.162	0.211	0.246	0.238	0.216	0.251	0.163
United States	0.393	0.501	0.593	0.619	0.617	0.555	0.471	0.383

6. Unstandardized average number of Hospitalization, ICU and mortality rate throughout the Delta and Omicron period

Table S15. The unstandardized average number of daily hospital occupancy, daily ICU occupancy and mortality rate per 100000 inhabitants

Country	Average daily hospital occupancy per 100000 inhabitants (aHOSP)			Average daily ICU occupancy per 100000 inhabitants (aICU)			Average daily mortality rate per 100000 inhabitants (aMOR)		
	Delta	Omicron	aHOSP Change [a] (Rank)	Delta	Omicron	aICU Change [b] (Rank)	Delta	Omicron	aMOR Change [c] (Rank)
Australia	0.733	14.153	13.419 (10)	0.122	1.165	1.044 (13)	0.004	0.188	0.185 (7)
Belgium	2.989	25.932	22.943 (19)	0.955	3.349	2.394 (21)	0.025	0.275	0.25 (12)
Bulgaria	18.235	72.530	54.295 (29)	1.770	7.626	5.856 (28)	0.161	1.047	0.886 (29)
Canada	1.815	18.267	16.451 (14)	0.746	2.368	1.623 (17)	0.021	0.266	0.245 (11)
Czechia	0.481	26.793	26.312 (21)	0.122	3.435	3.312 (25)	0.019	0.396	0.377 (20)
Switzerland	3.489	20.999	17.509 (15)	0.909	2.762	1.853 (18)	0.022	0.211	0.189 (8)
Denmark	1.270	16.692	15.422 (13)	0.211	0.863	0.652 (9)	0.012	0.335	0.323 (18)
Spain	13.085	27.737	14.652 (12)	3.131	4.088	0.956 (11)	0.118	0.343	0.225 (10)
Estonia	4.052	34.692	30.64 (25)	0.433	1.481	1.048 (14)	0.024	0.465	0.441 (22)

Finland	1.020	11.740	10.72 (6)	0.186	0.836	0.65 (8)	0.015	0.205	0.19 (9)
France	12.473	41.559	29.086 (24)	2.064	5.225	3.161 (23)	0.080	0.376	0.297 (17)
United Kingdom	2.560	22.194	19.634 (16)	0.382	1.010	0.627 (7)	0.025	0.300	0.275 (15)
Ireland	2.678	14.588	11.91 (7)	0.537	1.665	1.128 (15)	0.030	0.163	0.133 (5)
Israel	1.675	24.598	22.923 (18)	0.205	2.101	1.895 (20)	0.012	0.308	0.296 (16)
Italy	4.176	30.135	25.96 (20)	0.460	2.325	1.865 (19)	0.042	0.500	0.458 (23)
Japan	14.906	13.175	-1.731 (2)	1.817	0.889	-0.928 (2)	0.029	0.070	0.041 (4)
Luxembourg	2.234	10.360	8.127 (5)	0.554	2.025	1.471 (16)	0.028	0.205	0.177 (6)
Latvia	3.099	50.488	47.389 (28)	0.951	5.062	4.111 (27)	0.062	0.677	0.615 (28)
Malta	5.131	17.131	11.999 (9)	0.408	0.958	0.55 (5)	0.069	0.433	0.363 (19)
Malaysia	28.800	8.833	-19.968 (1)	3.907	0.588	-3.319 (1)	0.357	0.054	-0.302 (1)
Netherlands	2.113	6.644	4.531 (3)	0.925	1.538	0.613 (6)	0.026	0.060	0.034 (2)
Portugal	6.546	18.515	11.969 (8)	1.455	1.528	0.073 (3)	0.072	0.340	0.267 (13)
Romania	5.222	42.359	37.137 (26)	0.486	3.777	3.29 (24)	0.043	0.422	0.378 (21)
Singapore	3.158	11.114	7.956 (4)	0.036	0.223	0.187 (4)	0.001	0.039	0.038 (3)
Serbia	18.987	47.748	28.76 (22)	0.877	1.870	0.993 (12)	0.164	0.628	0.464 (24)
Slovakia	1.503	39.819	38.315 (27)	0.514	4.510	3.996 (26)	0.010	0.534	0.524 (26)
Slovenia	2.793	31.614	28.821 (23)	0.615	6.627	6.012 (29)	0.027	0.583	0.556 (27)
Sweden	1.580	15.068	13.488 (11)	0.263	1.024	0.761 (10)	0.011	0.279	0.268 (14)
United States	12.456	34.210	21.754 (17)	3.263	6.297	3.034 (22)	0.134	0.632	0.498 (25)

Green: Tertile 1 (Low score)-Good performance; Grey: Tertile 2(Medium score) – Medium performance; Orange: Tertile 3 (High score)-Poor performance

[a] The change of average daily hospital occupancy per 100000 inhabitants (aHOSP Change) = aHOSP of Omicron period – aHOSP of Delta period

[b] The change of average daily ICU occupancy per 100000 inhabitants (aICU Change) = aICU of Omicron period – aICU of Delta period

[c] The change of average daily mortality rate per 100000 inhabitants (aMOR Change) = aMOR of Omicron period – aMOR of Delta period
